# Supplementary material for: Fine-Tuning of the Optical and Electrochemical Properties of Ruthenium(II) Complexes with 2-Arylbenzimidazoles and 4,4′-Dimethoxycarbonyl-2,2′-bipyridine
Source: Molecules. 2023 Sep 9;28(18):6541. doi: 10.3390/molecules28186541 (PMC10536653; doi:10.3390/molecules28186541)
Supplement: Supplementary file 1 [file molecules-28-06541-s001.zip › molecules-2562542-supplementary.pdf]

Contents

1. Luminescence 3

1.1 Excitation and emission spectra 3

Figure S1. Excitation spectra for complexes 1-4, CH_3CN degassed solutions, room temperature. 3

Figure S2. Emission spectra for complexes 1-4, CH_3CN degassed solutions, room temperature. 3

Figure S3. Normalized emission spectra recorded at room temperature and at 77 K 4

1.2 Decay curves 5

Figure S4. Decay curve and exponential fit for complexes 1-4, recorded at 77K. 5

Figure S5. Decay curve and exponential fit for complexes 1-4, recorded at room temperature. 5

2 DFT 6

Figure S6. Optimized ground-state geometries for the investigated compounds. 6

Table S1. Comparison of bond lengths obtained from X-ray diffraction study and calculated by the DFT method. 6

Table S2. Cartesian coordinates of ground state geometries of complexes 1-4. 7

3 Absorbance 17

Figure S7. Absorbance spectra of complexes **1-4** in CH_3CN solutions. 17

Figure S8. Decomposition into Gaussian components of absorbance spectra. 17

Table S3. Decomposition into Gaussian components of absorbance spectra. 18

4 Cyclic voltammetry 18

Fig. S9. CV of 5 mM solutions of complexes in 0.1M TBAP/CH_3CN in wide range of potentials. 18

5 NMR spectroscopy 19

Figure S10. ^{1}H NMR spectrum of **1** (600 MHz, 298 K, acetone-d6). 19

Figure S11. 13C NMR spectrum of 1 (151 MHz, 298 K, acetone-d6). 20

Figure S12. 1H NMR spectrum of 1 (600 MHz, 298 K, $CDCl_3$). 21

Figure S13. Aromatic region of COSY ^{1}H,^{1}H spectrum of **1**. 22

Table S4. Assignment of ^{1}H signals of complex **1**. 22

Figure S14. ^{1}H NMR spectrum of **2** (600 MHz, 298 K, acetone-d^6). 23

Figure S15. ^{13}C NMR spectrum of **2** (151 MHz, 298 K, acetone-d^6). 24

Figure S16. Aromatic region of COSY ^{1}H,^{1}H spectrum of **2**. 25

Table S5. Assignment of ^{1}H signals of complex **2**. 25

Figure S17. ^{1}H NMR spectrum of **3** (600 MHz, 298 K, acetone-d^6). 26

Figure S18. ^{13}C NMR spectrum of **3** (151 MHz, 298 K, acetone-d^6). 27

Figure S19. Aromatic region of COSY ^{1}H,^{1}H spectrum of **3**. 28

Figure S20. HMBC spectrum of **3**. 28
Table S6. Assignment of ^{1}H signals of complex **3**. 29
Table S7. Assignment of ^{13}C signals of complex **3**. 30
Figure S21. ^{1}H NMR spectrum of **4** (600 MHz, 298 K, acetone-d^{6}). 31
Figure S22. ^{13}C NMR spectrum of **4** (151 MHz, 298 K, acetone-d^{6}). 32
Figure S23. Aromatic region of COSY ^{1}H,^{1}H spectrum of **4**. 33
Table S8. Assignment of ^{1}H signals of complex **4**. 33
Figure S24. ^{1}H spectrum of **L-NO$_2$** (1-benzyl-2-(4-nitrophenyl)benzimidazole) 34
Figure S25. ^{1}H spectrum of **L-H** (1-benzyl-2-phenylbenzimidazole) 35
Figure S26. ^{1}H spectrum of **L-OMe$_2$** (1-benzyl-2-(3,4-dimethoxyphenyl)benzimidazole) 35
Figure S27. ^{1}H spectrum of **L-NMe$_2$** (1-benzyl-2-(4-dimethylaminophenyl)benzimidazole) 36

1. **Luminescence**

1.1 Excitation and emission spectra

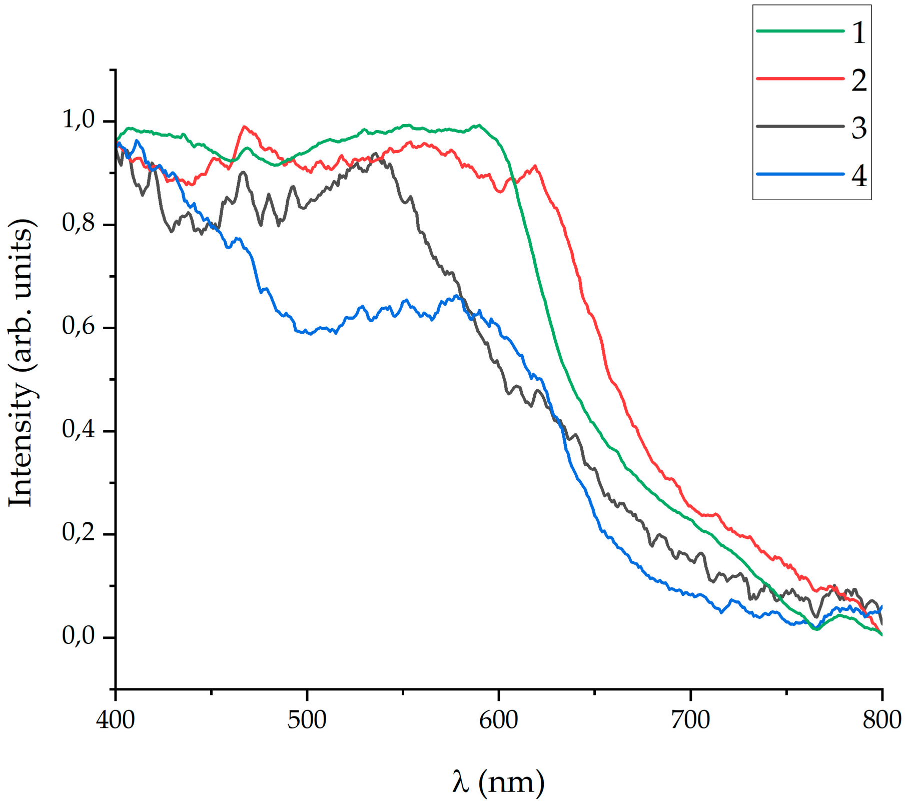

Figure S1. Excitation spectra for complexes 1-4, CH_3CN degassed solutions, room temperature.

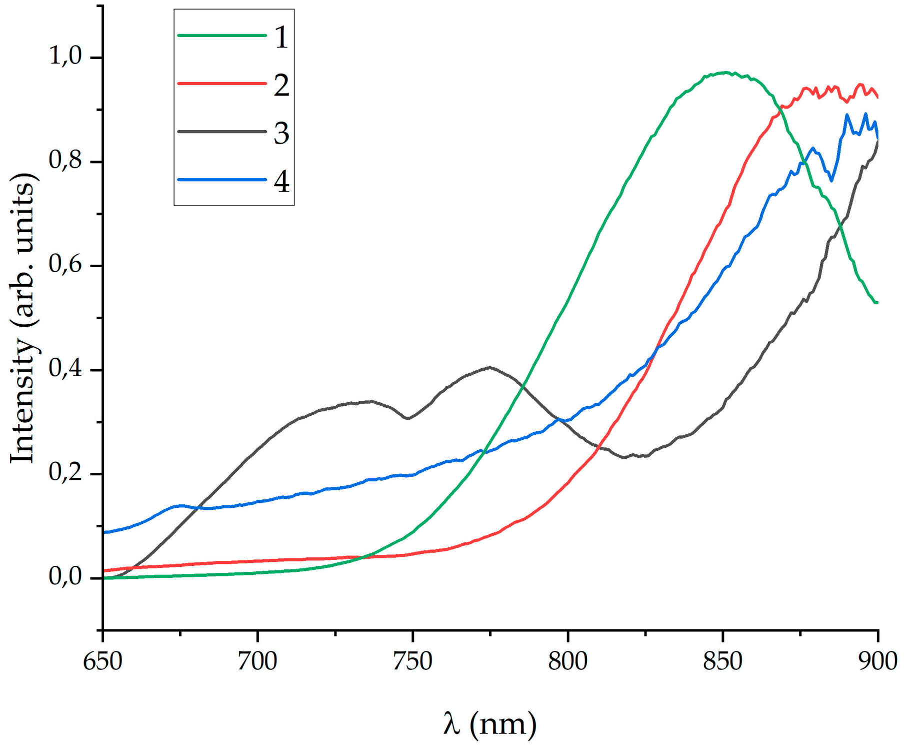

Figure S2. Emission spectra for complexes 1-4, CH_3CN degassed solutions, room temperature.

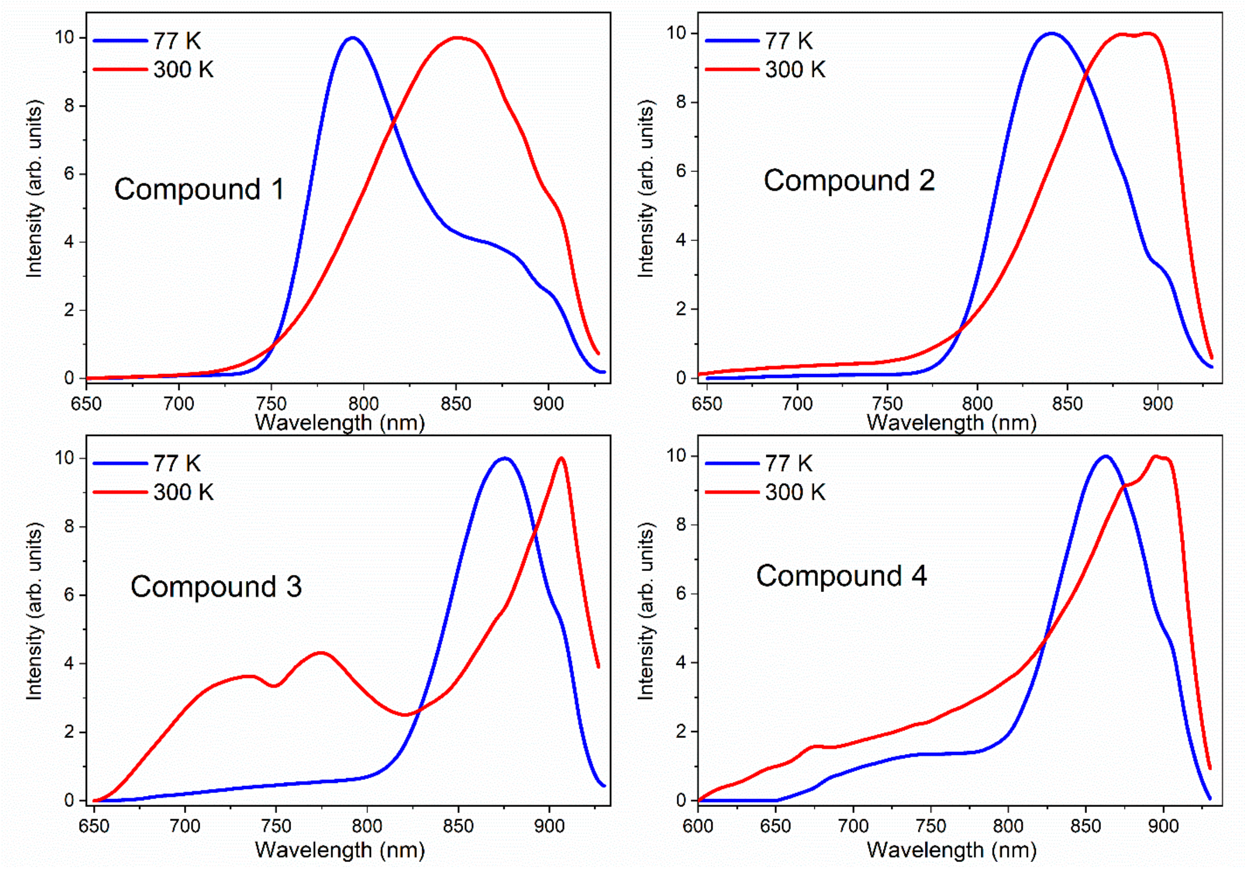

Figure S3. Normalized emission spectra recorded at room temperature and at 77 K; for complexes **3** and **4** it is observed that the ratio of intensities of the two emission bands increases with cooling.

1.2 Decay curves

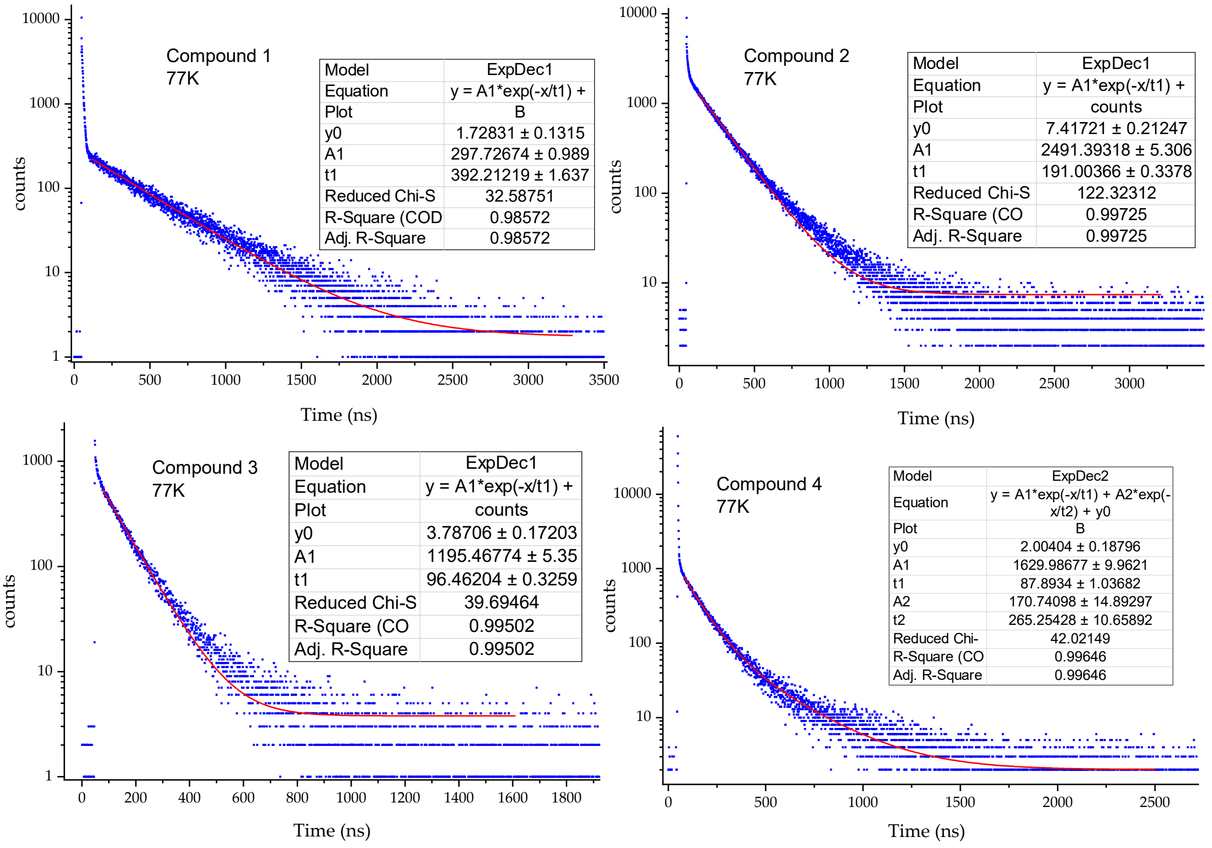

Figure S4. Decay curve and exponential fit for complexes 1-4, recorded at 77K.

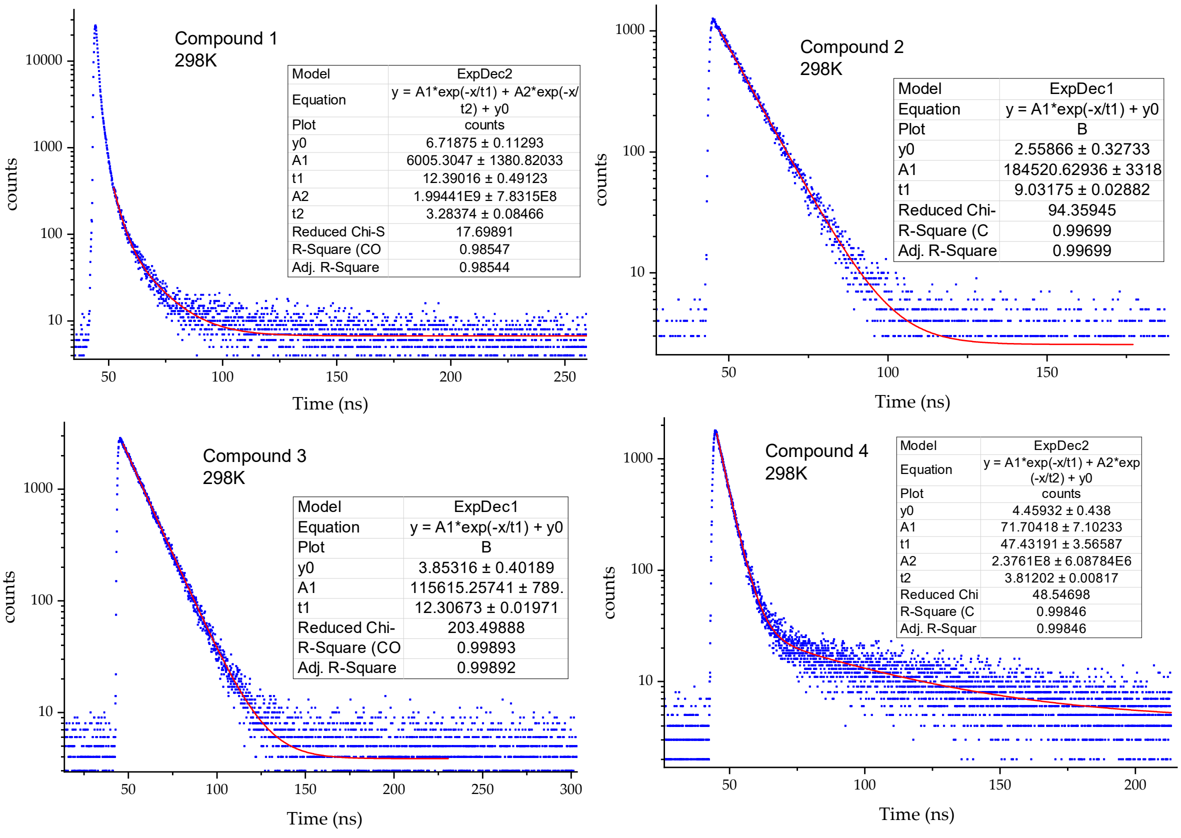

Figure S5. Decay curve and exponential fit for complexes 1-4, recorded at room temperature.

2 DFT

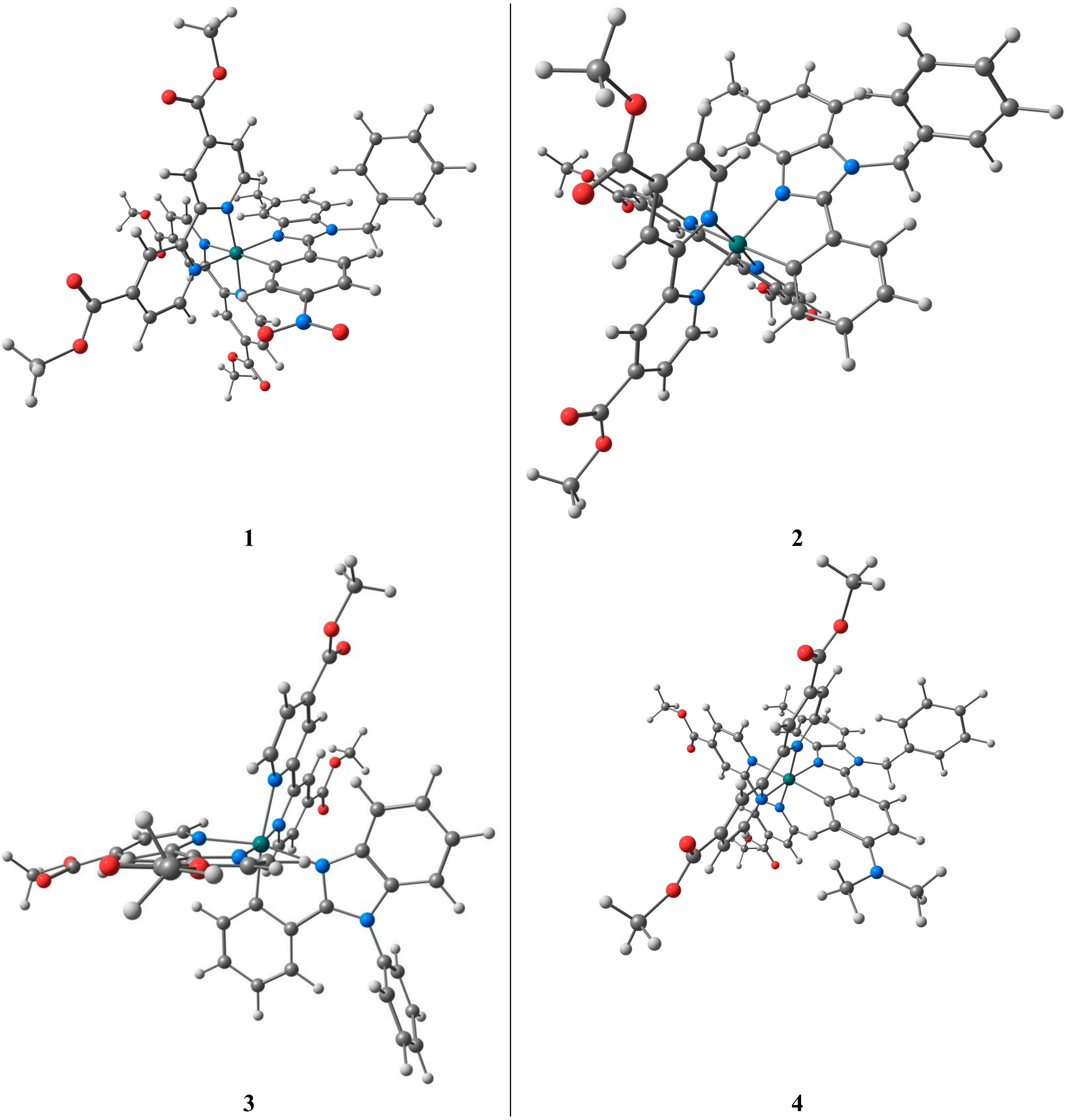

Figure S6. Optimized ground-state geometries for the investigated compounds.

Table S1. Comparison of bond lengths obtained from X-ray diffraction study and calculated by the DFT method.

Bond length, Å	**1**, X-ray data	**1**, DFT	**3**, X-ray data	**3**, DFT
Ru1-C1	2.056(6)	2.043	2.052(6)	2.049
Ru1-N1	2.047(5)	2.086	2.087(5)	2.092
Ru1-N3	2.034(4)	2.048	2.016(5)	2.041
Ru1-N4	2.023(4)	2.044	2.021(4)	2.035

Ru1-N5	2.055(4)	2.070	2.049(4)	2.070
Ru1-N6	2.115(4)	2.143	2.122(5)	2.143

Table S2. Cartesian coordinates of ground state geometries of complexes 1-4.

Cartesian coordinates of ground state geometry for the compound 1			
Ru	0.128874	0.153702	0.054167
O	-5.180015	4.620628	0.763265
O	-4.806024	1.730955	-4.762769
O	-3.584228	0.297969	-5.983783
O	5.871754	3.110908	-2.612857
N	1.467343	-1.202859	-0.683561
O	-4.308735	4.923650	2.809881
O	5.532464	-3.269147	-2.765930
N	1.549845	1.399135	-0.736443
O	4.761167	4.966173	-2.009364
N	-1.246157	1.587074	0.638510
O	4.283456	-5.025928	-2.140263
N	-1.496285	-2.446423	2.884288
N	-1.094937	0.369380	-1.692250
N	-1.035417	-1.254455	1.060378
C	1.191844	-1.145079	4.028645
H	0.810429	-1.890086	4.732426
C	2.652668	0.782865	-1.240999
C	0.569006	-0.912057	2.791472
C	-3.866066	0.990969	-4.885932
C	-3.234069	2.807830	0.061534
H	-4.017725	3.050527	-0.661138
C	-1.263846	2.180067	1.846810
H	-0.454105	1.900128	2.530476
C	2.848964	0.472181	3.462399
C	-2.465345	-2.759184	1.941812
C	2.597892	-0.679962	-1.230351
C	1.102574	0.012662	1.844733
C	-0.641525	-1.552930	2.302369
C	3.740192	1.516632	-1.707644
H	4.636695	1.030613	-2.106359
C	-2.868719	0.747298	-3.799497
C	2.261691	0.701811	2.222975
H	2.741998	1.437432	1.567132
C	-4.290410	4.401302	1.727877
C	-2.135677	1.224497	-1.556032
C	-3.246102	3.417330	1.312121
C	3.598106	-1.501744	-1.743274
H	4.511445	-1.090869	-2.185976

C	-2.235965	3.094460	2.219528
H	-2.223375	3.562489	3.208938
C	-3.042887	1.423899	-2.595890
H	-3.897732	2.101218	-2.503234
C	1.321339	-2.538722	-0.643606
H	0.391474	-2.906689	-0.193708
C	3.706744	2.906161	-1.657065
C	1.514199	2.741970	-0.707930
H	0.604089	3.192619	-0.294614
C	3.444760	-2.883874	-1.699467
C	2.341297	-0.447050	4.374589
H	2.849887	-0.607018	5.328226
C	-1.774500	-0.111102	-3.938282
H	-1.600118	-0.649444	-4.873888
C	4.541191	-3.726958	-2.262992
C	-2.168381	-1.993818	0.799420
C	-0.916404	-0.264910	-2.858947
H	-0.049562	-0.936284	-2.917736
C	2.564809	3.530937	-1.149490
H	2.502079	4.621069	-1.094602
C	-2.225770	1.897266	-0.253446
C	2.278756	-3.410888	-1.137985
H	2.122976	-4.491971	-1.086653
C	4.904049	3.652723	-2.149000
C	-4.473230	0.468302	-7.081637
H	-4.091515	-0.171022	-7.890148
H	-4.491841	1.524463	-7.400702
H	-5.496002	0.165343	-6.799133
C	-3.548091	-3.639033	1.969640
H	-3.776652	-4.254631	2.846528
C	-2.967143	-2.089706	-0.346890
H	-2.735541	-1.502831	-1.242376
C	-6.217530	5.546182	1.064627
H	-6.847436	5.607629	0.165905
H	-5.792352	6.535169	1.307089
H	-6.808614	5.193956	1.927344
C	-4.329353	-3.716027	0.825664
H	-5.188759	-4.397996	0.813893
C	-4.057986	-2.953489	-0.333997
C	5.858219	5.766372	-2.433677
H	6.054944	5.611165	-3.508382
H	6.766808	5.504330	-1.865041
H	5.571818	6.809946	-2.241033
C	5.274676	-5.911870	-2.646122
H	5.423243	-5.747120	-3.727149

H	4.903593	-6.929686	-2.459734
H	6.235706	-5.750278	-2.128456
C	-4.947165	-3.089039	-1.533581
H	-4.611029	-2.444004	-2.365189
H	-4.972220	-4.131452	-1.903842
H	-5.991630	-2.812258	-1.294895
C	-1.397359	-3.074566	4.186132
H	-1.190462	-2.299901	4.946986
H	-2.407980	-3.453329	4.428951
C	-0.383219	-4.189800	4.253469
C	0.090885	-4.602090	5.502466
C	0.075512	-4.836518	3.103928
C	1.004523	-5.647669	5.602414
C	0.992712	-5.882088	3.203535
C	1.458195	-6.290596	4.451073
H	-0.260252	-4.094063	6.411094
H	-0.287761	-4.524430	2.115221
H	1.369572	-5.960003	6.587094
H	1.345763	-6.383953	2.295320
H	2.179647	-7.111456	4.528070
N	4.069925	1.225913	3.820014
O	4.566897	0.999376	4.899532
O	4.491273	2.021882	3.008413
Cartesian coordinates of ground state geometry for the compound 2			
Ru	0.363203	0.223598	0.167110
O	-4.962630	4.648217	1.003396
O	-4.677281	1.786068	-4.549011
O	-3.473362	0.359465	-5.794647
O	5.995358	3.180038	-2.729626
N	1.691758	-1.133729	-0.574984
O	-4.034199	4.972463	3.021117
O	5.755766	-3.213182	-2.652223
N	1.759926	1.463223	-0.662567
O	4.857926	5.036123	-2.183040
N	-1.002639	1.654161	0.777763
O	4.517094	-4.965273	-1.994649
N	-1.239618	-2.356799	3.023457
N	-0.899010	0.437469	-1.554579
N	-0.791428	-1.183858	1.185869
C	1.455376	-1.047471	4.139559
H	1.077984	-1.788698	4.850410
C	2.853665	0.847209	-1.188410
C	0.824964	-0.825777	2.904257
C	-3.736210	1.050574	-4.690264

C	-3.014779	2.858202	0.250302
H	-3.818715	3.093218	-0.452390
C	-0.994527	2.247389	1.986257
H	-0.166246	1.971599	2.649427
C	3.131485	0.580876	3.563035
C	-2.214952	-2.681244	2.089657
C	2.812688	-0.614416	-1.145945
C	1.358192	0.092150	1.952168
C	-0.387317	-1.469048	2.428202
C	3.914434	1.581976	-1.712519
H	4.801584	1.095098	-2.130709
C	-2.716439	0.810640	-3.624662
C	2.519574	0.784384	2.325118
H	2.975744	1.510697	1.636275
C	-4.041543	4.443242	1.942069
C	-1.941545	1.285862	-1.394812
C	-3.000339	3.468225	1.500656
C	3.813009	-1.438710	-1.654410
H	4.718363	-1.029644	-2.114858
C	-1.964491	3.154116	2.382155
H	-1.931063	3.622056	3.371074
C	-2.870903	1.482563	-2.415736
H	-3.727818	2.154311	-2.303675
C	1.556285	-2.470226	-0.508733
H	0.635006	-2.835854	-0.040181
C	3.862150	2.971820	-1.703876
C	1.707272	2.806735	-0.670778
H	0.805658	3.256494	-0.238222
C	3.671016	-2.820684	-1.581828
C	2.605962	-0.340980	4.468821
C	-1.619756	-0.040798	-3.787929
H	-1.460960	-0.575617	-4.728297
C	4.767856	-3.666409	-2.137690
C	-1.926082	-1.926877	0.938376
C	-0.739815	-0.192660	-2.726286
H	0.129289	-0.859546	-2.802669
C	2.728582	3.595897	-1.174180
H	2.649479	4.686211	-1.153016
C	-2.006749	1.956348	-0.089649
C	2.514283	-3.344302	-0.997403
H	2.366685	-4.425168	-0.922717
C	5.026575	3.719412	-2.264264
C	-4.385628	0.525647	-6.873247
H	-4.016845	-0.110197	-7.690535
H	-4.417840	1.582047	-7.190603

H	-5.400570	0.215811	-6.570515
C	-3.294862	-3.562203	2.134175
H	-3.514620	-4.169463	3.019244
C	-2.732997	-2.036692	-0.200167
H	-2.509529	-1.459761	-1.104085
C	-6.000467	5.563145	1.332074
H	-6.660566	5.610257	0.454323
H	-5.580167	6.559283	1.553173
H	-6.558613	5.210777	2.216441
C	-4.085790	-3.653205	0.996554
H	-4.943941	-4.336910	0.998334
C	-3.823329	-2.902491	-0.171121
C	5.919161	5.837071	-2.687358
H	6.081773	5.631042	-3.759249
H	6.854309	5.625715	-2.140875
H	5.614854	6.882832	-2.538457
C	5.509619	-5.853380	-2.492110
H	5.652595	-5.704180	-3.576208
H	5.144443	-6.870245	-2.289210
H	6.472645	-5.680185	-1.981802
C	-4.718941	-3.050870	-1.364805
H	-4.393991	-2.405504	-2.200762
H	-4.736113	-4.094589	-1.732127
H	-5.764677	-2.783287	-1.121175
C	-1.140455	-2.963603	4.333925
H	-0.913487	-2.179453	5.078980
H	-2.155023	-3.321741	4.592079
C	-0.145814	-4.095336	4.415366
C	0.306190	-4.512330	5.671031
C	0.316262	-4.752728	3.273529
C	1.200254	-5.573121	5.785065
C	1.213905	-5.813820	3.387038
C	1.656790	-6.227229	4.641065
H	-0.047610	-3.995518	6.573653
H	-0.028836	-4.435831	2.280011
H	1.547386	-5.889293	6.775085
H	1.569496	-6.323892	2.484376
H	2.362500	-7.060667	4.728982
H	4.038718	1.140871	3.820779
H	3.097687	-0.516536	5.431454
Cartesian coordinates of ground state geometry for the compound 3			
Ru	0.241892	0.153697	-0.043387
O	-5.138442	4.466609	0.988680
O	-4.929648	1.700330	-4.616723

O	-3.714265	0.338429	-5.922033
O	5.892286	3.175501	-2.831829
N	1.558954	-1.182989	-0.829679
O	-4.159778	4.790003	2.982532
O	5.580230	-3.220045	-3.031871
N	1.643932	1.412597	-0.828768
O	4.770334	5.016978	-2.209571
N	-1.121329	1.565328	0.615602
O	4.328589	-4.983340	-2.431820
N	-1.419647	-2.402872	2.801229
N	-1.042829	0.396614	-1.741724
N	-0.921377	-1.278411	0.943324
C	1.296867	-1.177637	3.914112
H	0.876336	-1.904275	4.610831
C	2.731596	0.808812	-1.381328
C	0.668580	-0.918632	2.682195
C	-3.970354	0.997340	-4.796177
C	-3.172588	2.731269	0.158687
H	-4.001320	2.956316	-0.517957
C	-1.088070	2.149877	1.827936
H	-0.233279	1.887187	2.462276
C	3.056779	0.379909	3.348336
C	-2.372870	-2.749960	1.851046
C	2.678479	-0.652409	-1.393992
C	1.232820	-0.016423	1.742411
C	-0.545726	-1.537213	2.201713
C	3.797870	1.554761	-1.877706
H	4.679335	1.077024	-2.317836
C	-2.918381	0.765212	-3.761030
C	2.418666	0.616419	2.124804
H	2.932495	1.333335	1.468128
C	-4.185404	4.272938	1.897754
C	-2.105423	1.210365	-1.538423
C	-3.135145	3.328198	1.414850
C	3.665665	-1.465773	-1.944271
H	4.568779	-1.046772	-2.400142
C	-2.065141	3.031331	2.261059
H	-2.011302	3.492473	3.252249
C	-3.064297	1.402611	-2.532624
H	-3.937919	2.045073	-2.383859
C	1.414024	-2.520712	-0.808011
H	0.496485	-2.895223	-0.338802
C	3.759241	2.943292	-1.810253
C	1.605816	2.755700	-0.777829
H	0.709675	3.195518	-0.324626

C	3.511871	-2.848105	-1.922351
C	2.482075	-0.541220	4.261431
C	-1.799669	-0.046706	-3.970670
H	-1.645761	-0.552301	-4.927855
C	4.592406	-3.681914	-2.524560
C	-2.052045	-2.025120	0.688386
C	-0.891720	-0.198345	-2.932994
H	-0.004851	-0.836072	-3.045793
C	2.633880	3.555188	-1.249569
H	2.566257	4.644217	-1.180181
C	-2.154810	1.856319	-0.220460
C	2.357706	-3.383251	-1.341958
H	2.201266	-4.464835	-1.306681
C	4.928442	3.702962	-2.342845
C	-4.657639	0.499631	-6.973921
H	-4.291015	-0.107731	-7.813590
H	-4.729092	1.560924	-7.267786
H	-5.655201	0.153329	-6.653348
C	-3.459271	-3.621173	1.892741
H	-3.703956	-4.202915	2.788523
C	-2.830711	-2.162242	-0.465922
H	-2.579773	-1.611337	-1.379194
C	-6.187089	5.352760	1.358090
H	-6.875594	5.391047	0.501955
H	-5.784611	6.357220	1.574800
H	-6.708369	4.979606	2.256341
C	-4.223479	-3.739091	0.737932
H	-5.085536	-4.417891	0.736872
C	-3.926771	-3.022535	-0.441614
C	5.836623	5.828785	-2.683812
H	5.995531	5.664900	-3.763557
H	6.771316	5.588588	-2.148534
H	5.541069	6.870180	-2.492459
C	5.303717	-5.860330	-2.979755
H	5.437896	-5.663734	-4.057412
H	4.927208	-6.880705	-2.819400
H	6.274425	-5.723337	-2.472854
C	-4.790566	-3.200162	-1.654841
H	-4.454530	-2.561397	-2.491537
H	-4.782953	-4.248707	-2.008861
H	-5.845875	-2.945455	-1.440810
C	-1.424235	-2.868536	4.170339
H	-1.239555	-2.005651	4.838854
H	-2.459350	-3.189955	4.392745
C	-0.453998	-3.984722	4.468561

C	-0.227016	-4.332060	5.804606
C	0.225715	-4.671421	3.461956
C	0.663344	-5.351259	6.130154
C	1.122937	-5.688659	3.787733
C	1.344328	-6.030985	5.119591
H	-0.758168	-3.793168	6.601693
H	0.060171	-4.405741	2.409133
H	0.828781	-5.617107	7.180395
H	1.653739	-6.220366	2.989923
H	2.048191	-6.831590	5.372740
O	4.171383	1.090533	3.581862
C	5.259955	0.575786	4.318169
H	5.103168	0.665290	5.406019
H	5.448018	-0.485174	4.066569
H	6.137800	1.174709	4.024268
O	3.119507	-0.741015	5.438740
C	2.659551	-1.752436	6.288209
H	1.643244	-1.533702	6.677658
H	2.637910	-2.739230	5.778680
H	3.359022	-1.799421	7.137705
Cartesian coordinates of ground state geometry for the compound 4			
Ru	0.160562	0.195937	0.016065
O	-5.198516	4.562474	0.925871
O	-4.914605	1.758428	-4.662530
O	-3.707043	0.348993	-5.923819
O	5.919811	3.155872	-2.603658
N	1.490833	-1.159955	-0.718709
O	-4.247448	4.894119	2.931621
O	5.608714	-3.245904	-2.681366
N	1.573655	1.434591	-0.779869
O	4.748845	5.010129	-2.127195
N	-1.204001	1.620089	0.639874
O	4.322541	-4.995242	-2.114684
N	-1.452877	-2.407078	2.843680
N	-1.106185	0.421730	-1.700181
N	-1.003349	-1.220363	1.013826
C	1.242376	-1.081318	3.975477
H	0.874309	-1.821029	4.693783
C	2.687473	0.818936	-1.263799
C	0.598838	-0.872230	2.743284
C	-3.968060	1.031809	-4.813407
C	-3.234030	2.805476	0.139168
H	-4.047474	3.035499	-0.554174
C	-1.189788	2.206016	1.851919

H	-0.351627	1.934321	2.504489
C	2.947976	0.560265	3.416342
C	-2.422388	-2.733172	1.902358
C	2.635073	-0.642343	-1.243357
C	1.148924	0.052183	1.805858
C	-0.600456	-1.510394	2.259249
C	3.776012	1.554448	-1.727003
H	4.679178	1.066978	-2.108722
C	-2.939468	0.794287	-3.756253
C	2.301784	0.745746	2.167963
H	2.732127	1.464297	1.462109
C	-4.261681	4.367061	1.851375
C	-2.156453	1.257825	-1.526861
C	-3.214126	3.407307	1.393354
C	3.644790	-1.467909	-1.730699
H	4.568878	-1.060198	-2.153628
C	-2.165672	3.099508	2.262461
H	-2.128097	3.561378	3.254047
C	-3.093760	1.454011	-2.540671
H	-3.956879	2.115755	-2.417259
C	1.339768	-2.496128	-0.680413
H	0.399538	-2.858882	-0.248559
C	3.730760	2.944485	-1.702742
C	1.526580	2.778995	-0.778452
H	0.606338	3.227699	-0.385818
C	3.487355	-2.849342	-1.686366
C	2.387609	-0.383671	4.312270
H	2.858294	-0.588263	5.277007
C	-1.834654	-0.044100	-3.933480
H	-1.675901	-0.569024	-4.879395
C	4.594670	-3.696484	-2.217238
C	-2.132367	-1.969146	0.757049
C	-0.947101	-0.196177	-2.878433
H	-0.071034	-0.852632	-2.965073
C	2.574003	3.568356	-1.223180
H	2.497250	4.658732	-1.196528
C	-2.219132	1.917357	-0.216316
C	2.305595	-3.370995	-1.151508
H	2.144784	-4.451379	-1.101210
C	4.923747	3.692738	-2.195877
C	-4.628850	0.512591	-6.994223
H	-4.261062	-0.116001	-7.817602
H	-4.672901	1.570194	-7.306238
H	-5.638794	0.192472	-6.685426
C	-3.494680	-3.621473	1.936451

H	-3.713085	-4.235260	2.817510
C	-2.933436	-2.079985	-0.384649
H	-2.710447	-1.497391	-1.285111
C	-6.244089	5.461483	1.271795
H	-6.918624	5.499759	0.404599
H	-5.835421	6.463704	1.487385
H	-6.783188	5.100300	2.164413
C	-4.281766	-3.713146	0.794216
H	-5.134607	-4.403466	0.788872
C	-4.018835	-2.954638	-0.366066
C	5.832795	5.811216	-2.579052
H	6.052566	5.600038	-3.639706
H	6.738937	5.606024	-1.983404
H	5.518342	6.856996	-2.452613
C	5.322343	-5.884451	-2.594085
H	5.512610	-5.709417	-3.667012
H	4.935227	-6.900822	-2.433568
H	6.265776	-5.739217	-2.040173
C	-4.907021	-3.100597	-1.566009
H	-4.575478	-2.455146	-2.399464
H	-4.924044	-4.143846	-1.934735
H	-5.953979	-2.830824	-1.329692
C	-1.350727	-3.017522	4.149965
H	-1.125176	-2.234872	4.897599
H	-2.362913	-3.381860	4.409636
C	-0.350760	-4.145089	4.229171
C	0.086428	-4.578076	5.484750
C	0.132111	-4.782428	3.084719
C	0.985872	-5.634589	5.596009
C	1.035286	-5.839168	3.195227
C	1.463215	-6.268561	4.449062
H	-0.284357	-4.077317	6.389664
H	-0.201187	-4.452018	2.091714
H	1.320384	-5.963855	6.586194
H	1.407072	-6.333141	2.290067
H	2.172909	-7.098929	4.534677
N	4.081603	1.258664	3.741983
C	4.664101	2.172451	2.790774
H	3.968708	2.994187	2.521836
H	5.566847	2.629479	3.224948
H	4.963613	1.662719	1.851023
C	4.725941	1.037317	5.015162
H	4.047966	1.256966	5.863799
H	5.084516	-0.006230	5.127763
H	5.598385	1.702555	5.106847

3 Absorbance

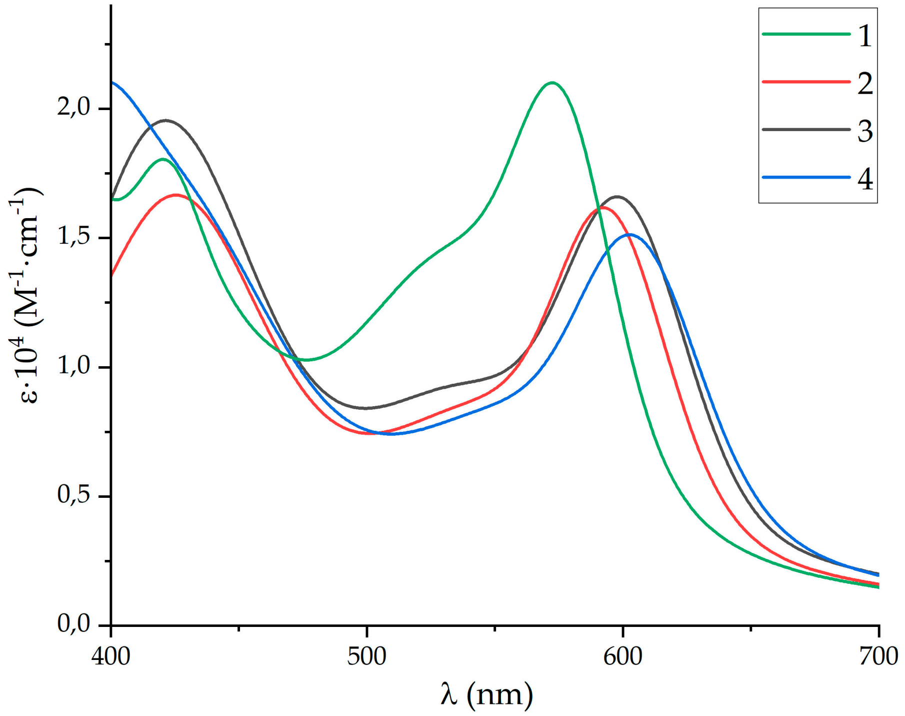

Figure S7. Absorbance spectra of complexes **1-4** in CH_3CN solutions.

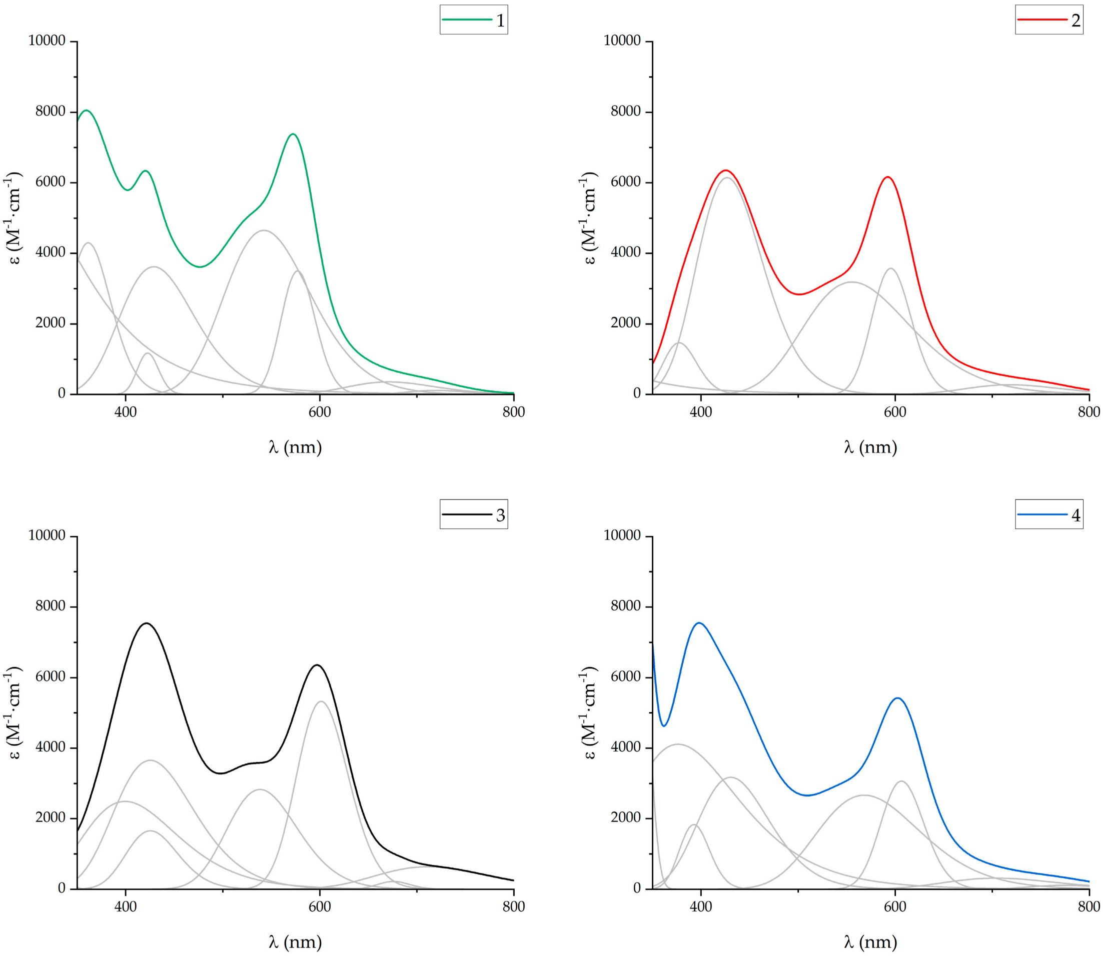

Figure S8. Decomposition into Gaussian components of absorbance spectra.

Table S3. Decomposition into Gaussian components of absorbance spectra.

Complex	λ_{abs} of gaussians, nm
**1**	429, 542, 577, 671, 719
**2**	427, 555, 596, 718, 760
**3**	425, 538, 601, 675, 711
**4**	431, 568, 606, 704, 773

4 Cyclic voltammetry

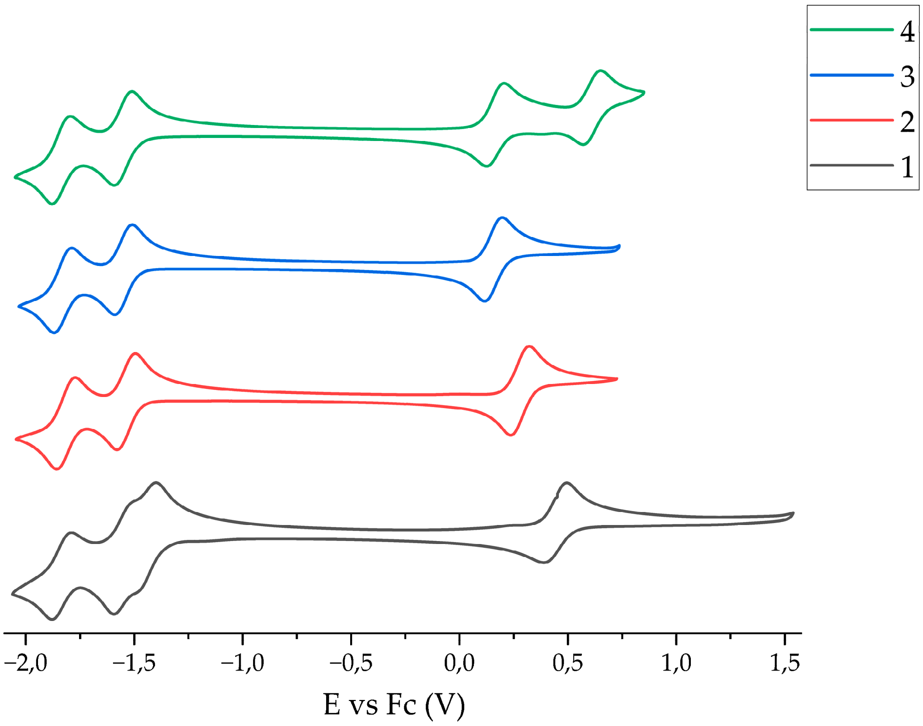

Fig. S9. CV of 5 mM solutions of complexes in 0.1M TBAP/CH_3CN in wide range of potentials.

5 NMR spectroscopy

Complex 1, acetone-d6

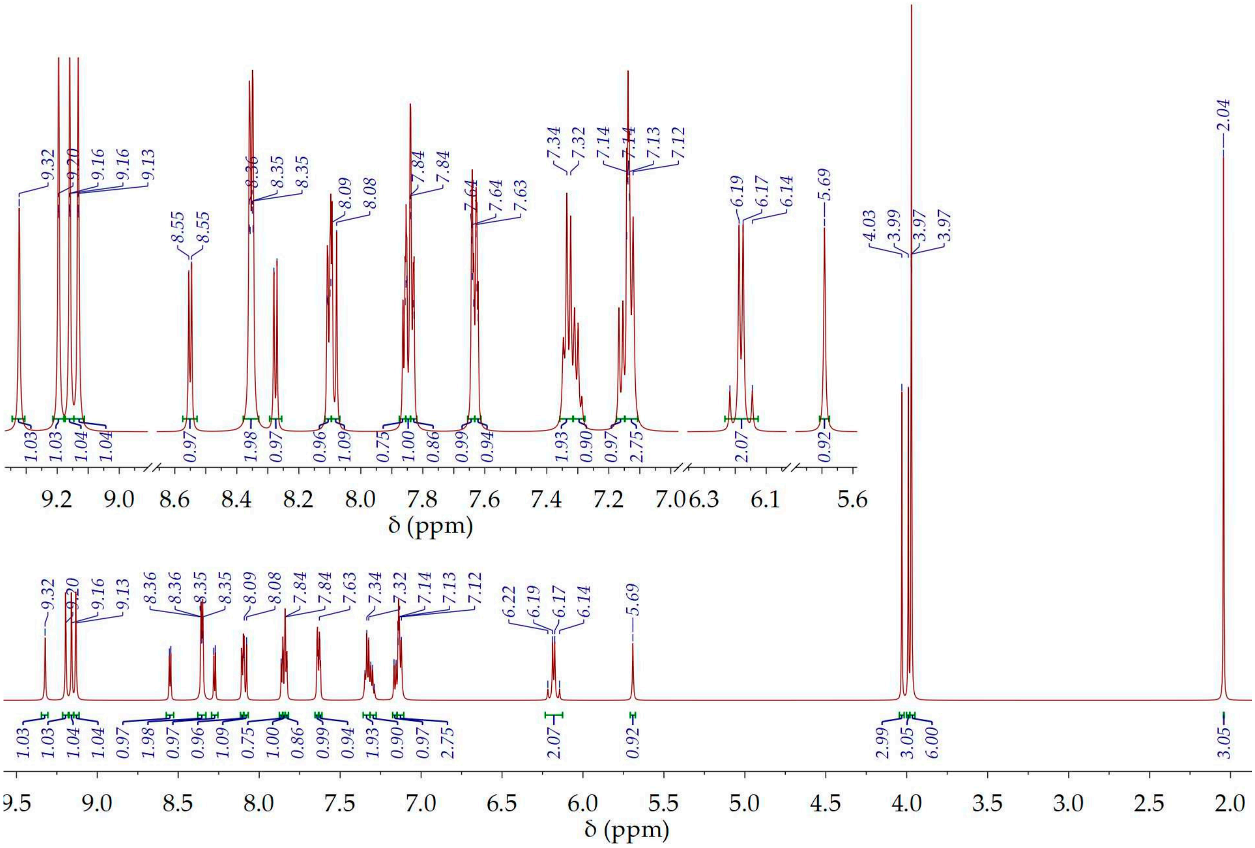

Figure S10. ^{1}H NMR spectrum of **1** (600 MHz, 298 K, acetone-d6).

^{1}H NMR (600 MHz, Acetone-d_6) δ 9.32 (s, 1H), 9.21 – 9.18 (m, 1H), 9.17 – 9.15 (m, 1H), 9.15 – 9.11 (m, 1H), 8.55 (d, *J* = 5.3 Hz, 1H), 8.35 (dd, *J* = 5.9, 1.6 Hz, 2H), 8.28 (d, *J* = 6.0 Hz, 1H), 8.10 (dd, *J* = 5.7, 1.6 Hz, 1H), 8.09 (d, *J* = 8.7 Hz, 1H), 7.86 (d, *J* = 4.2 Hz, 1H), 7.85 – 7.84 (m, 1H), 7.84 – 7.82 (m, 1H), 7.66 – 7.63 (m, 1H), 7.63 – 7.61 (m, 1H), 7.34 (t, *J* = 7.2 Hz, 2H), 7.30 (d, *J* = 7.0 Hz, 1H), 7.16 (d, *J* = 7.8 Hz, 1H), 7.15 – 7.11 (m, 3H), 6.23 – 6.13 (m, 2H), 5.69 (s, 1H), 4.03 (s, 3H), 3.99 (s, 3H), 3.98 – 3.95 (m, 6H), 2.04 (s, 3H).

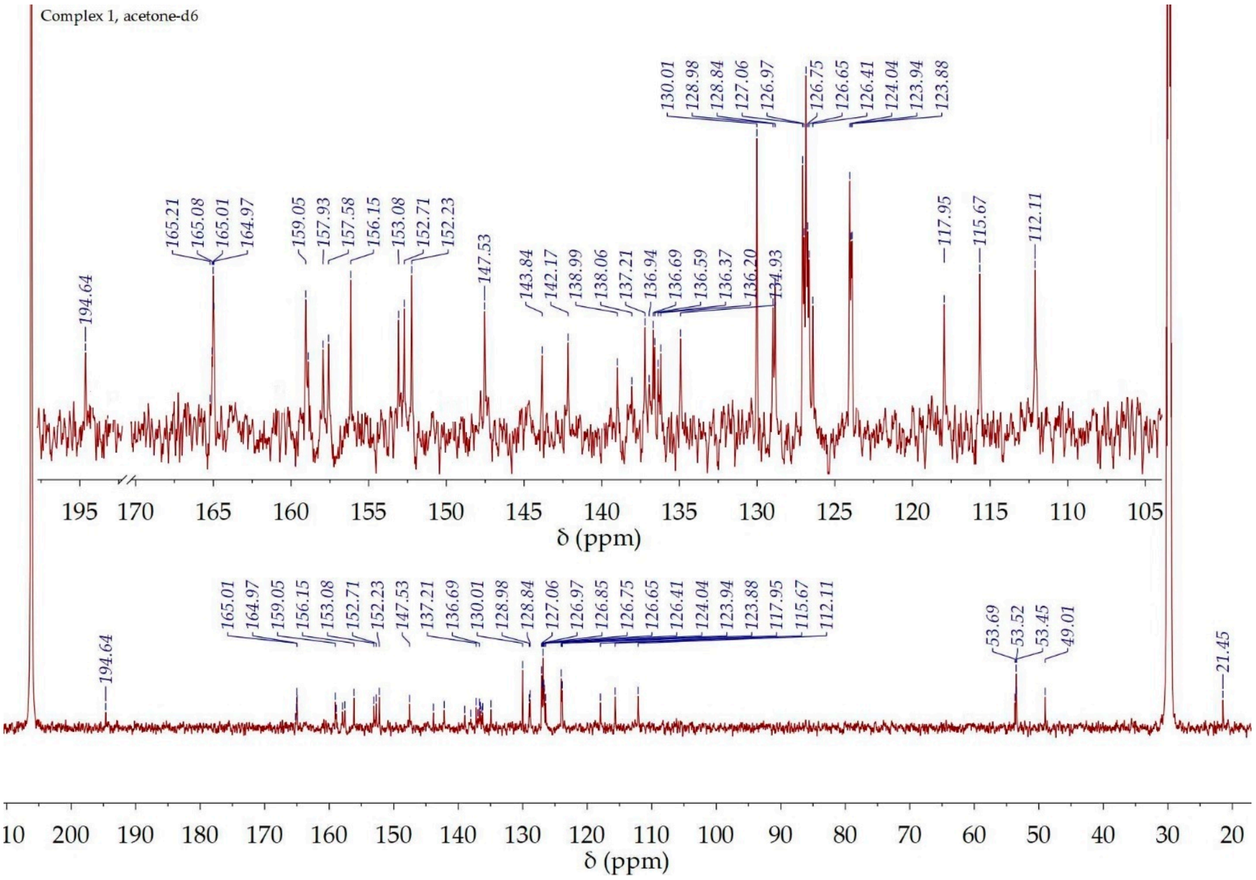

Figure S11. 13C NMR spectrum of 1 (151 MHz, 298 K, acetone-d6).

^{13}C NMR (151 MHz, Acetone-d^6) δ 194.64, 165.21, 165.08, 165.01, 164.97, 159.05, 158.89, 157.93, 157.58, 156.15, 153.08, 152.71, 152.23, 147.53, 143.84, 142.17, 138.99, 138.06, 137.21, 136.94, 136.69, 136.59, 136.37, 136.20, 134.93, 130.01, 130.01, 128.98, 128.84, 127.06, 126.97, 126.85, 126.85, 126.75, 126.65, 126.41, 124.04, 124.04, 123.94, 123.88, 117.95, 115.67, 112.11, 53.69, 53.52, 53.52, 53.45, 49.01, 21.45.

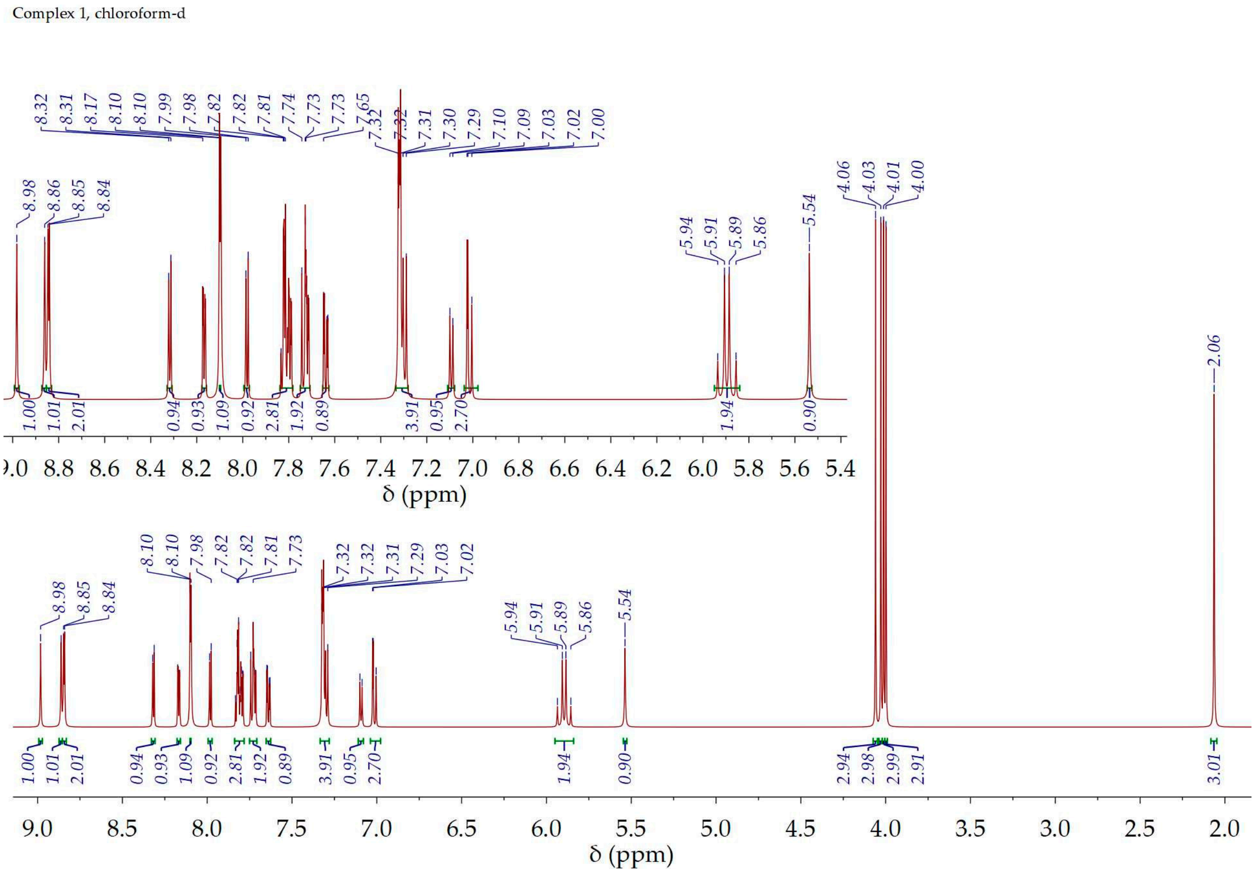

Figure S12. 1H NMR spectrum of 1 (600 MHz, 298 K, $CDCl_3$).

^{1}H NMR (600 MHz, Chloroform-*d*) δ 8.99 – 8.97 (m, 1H), 8.87 – 8.86 (m, 1H), 8.85 – 8.83 (m, 2H), 8.32 (d, *J* = 5.7 Hz, 1H), 8.17 (dd, *J* = 5.6, 1.6 Hz, 1H), 8.11 – 8.10 (m, 1H), 7.98 (d, *J* = 6.0 Hz, 1H), 7.83 (dd, *J* = 6.0, 1.6 Hz, 1H), 7.81 (s, 1H), 7.81 – 7.79 (m, 1H), 7.74 (d, *J* = 8.7 Hz, 1H), 7.72 (dd, *J* = 6.0, 1.8 Hz, 1H), 7.64 (dd, *J* = 8.7, 2.4 Hz, 1H), 7.34 – 7.31 (m, 3H), 7.30 (d, *J* = 8.5 Hz, 1H), 7.11 – 7.08 (m, 1H), 7.02 (d, *J* = 2.3 Hz, 1H), 7.00 (dd, *J* = 5.4, 3.9 Hz, 2H), 5.94 – 5.85 (m, 2H), 5.54 (s, 1H), 4.06 (s, 3H), 4.03 (s, 3H), 4.01 (s, 3H), 4.00 (s, 3H), 2.06 (s, 3H).

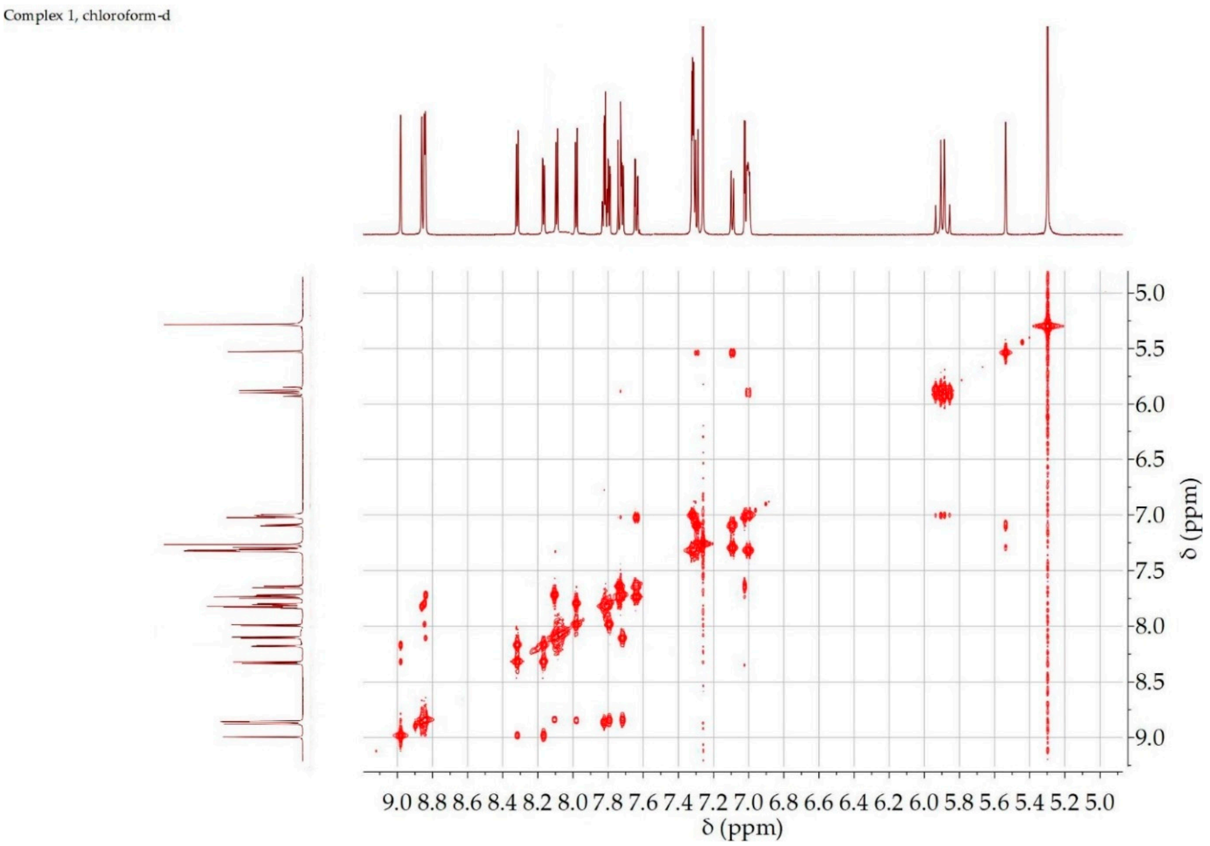

Figure S13. Aromatic region of COSY ^1H,^1H spectrum of **1**.

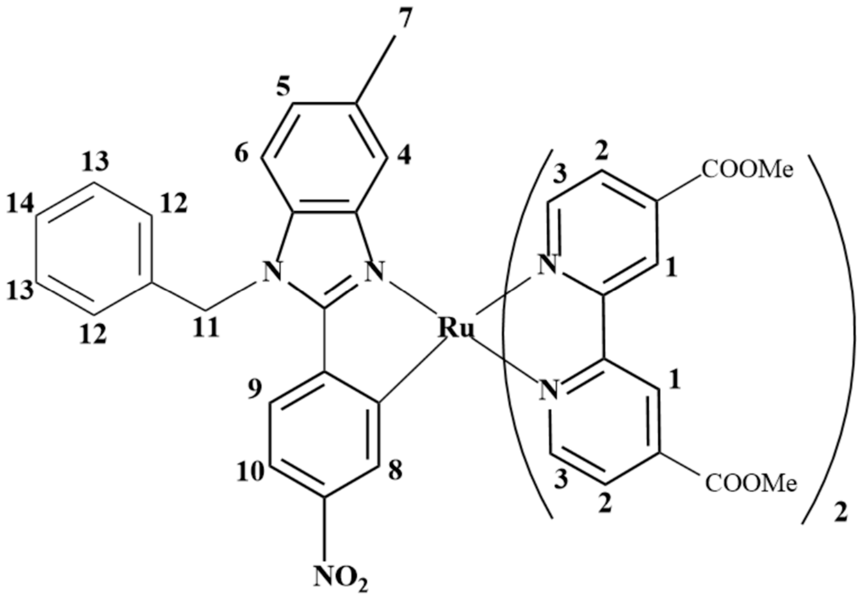

Table S4. Assignment of ^1H signals of complex **1**.

Hydrogen	^1H δ, ppm (acetone-d_6)	^1H δ, ppm (chloroform-d)
**1**	9.32 (s, 1H), 9.21 – 9.18 (m, 1H), 9.17 – 9.15 (m, 1H), 9.15 – 9.11 (m, 1H)	8.99 – 8.97 (m, 1H), 8.87 – 8.86 (m, 1H), 8.85 – 8.83 (m, 2H)
**2**	8.55 (d, J = 5.3 Hz, 1H), 8.28 (d, J = 6.0 Hz, 1H), 8.10 (dd, J = 5.7, 1.6 Hz, 1H), 8.09 (d, J = 8.7 Hz, 1H)	8.32 (d, J = 5.7 Hz, 1H), 8.11 – 8.10 (m, 1H), 7.98 (d, J = 6.0 Hz, 1H), 7.83 (dd, J = 6.0, 1.6 Hz, 1H)
**3**	8.35 (dd, J = 5.9, 1.6 Hz, 2H), 7.86 (d, J = 4.2 Hz, 1H), 7.85 – 7.84 (m, 1H)	8.17 (dd, J = 5.6, 1.6 Hz, 1H), 7.81 (s, 1H), 7.81 – 7.79 (m, 1H), 7.72 (dd, J =

		6.0, 1.8 Hz, 1H)
**4**	5.69 (s, 1H)	5.54 (s, 1H)
**5**	7.30 (d, J = 7.0 Hz, 1H)	7.11 – 7.08 (m, 1H)
**6**	7.63 – 7.61 (m, 1H)	7.30 (d, J = 8.5 Hz, 1H)
**7**	2.04 (s, 3H)	2.06 (s, 3H)
**8**	7.16 (d, J = 7.8 Hz, 1H)	7.02 (d, J = 2.3 Hz, 1H)
**9**	7.84 – 7.82 (m, 1H)	7.74 (d, J = 8.7 Hz, 1H)
**10**	7.66 – 7.63 (m, 1H)	7.64 (dd, J = 8.7, 2.4 Hz, 1H)
**11**	6.23 – 6.13 (m, 2H)	5.94 – 5.85 (m, 2H)
**12**	7.34 (t, J = 7.2 Hz, 2H)	7.00 (dd, J = 5.4, 3.9 Hz, 2H)
**13**	7.15 – 7.11 (m, 3H)	7.34 – 7.31 (m, 3H),
**14**		
**COOMe**	4.03 (s, 3H), 3.99 (s, 3H), 3.98 – 3.95 (m, 6H)	4.06 (s, 3H), 4.03 (s, 3H), 4.01 (s, 3H), 4.00 (s, 3H)

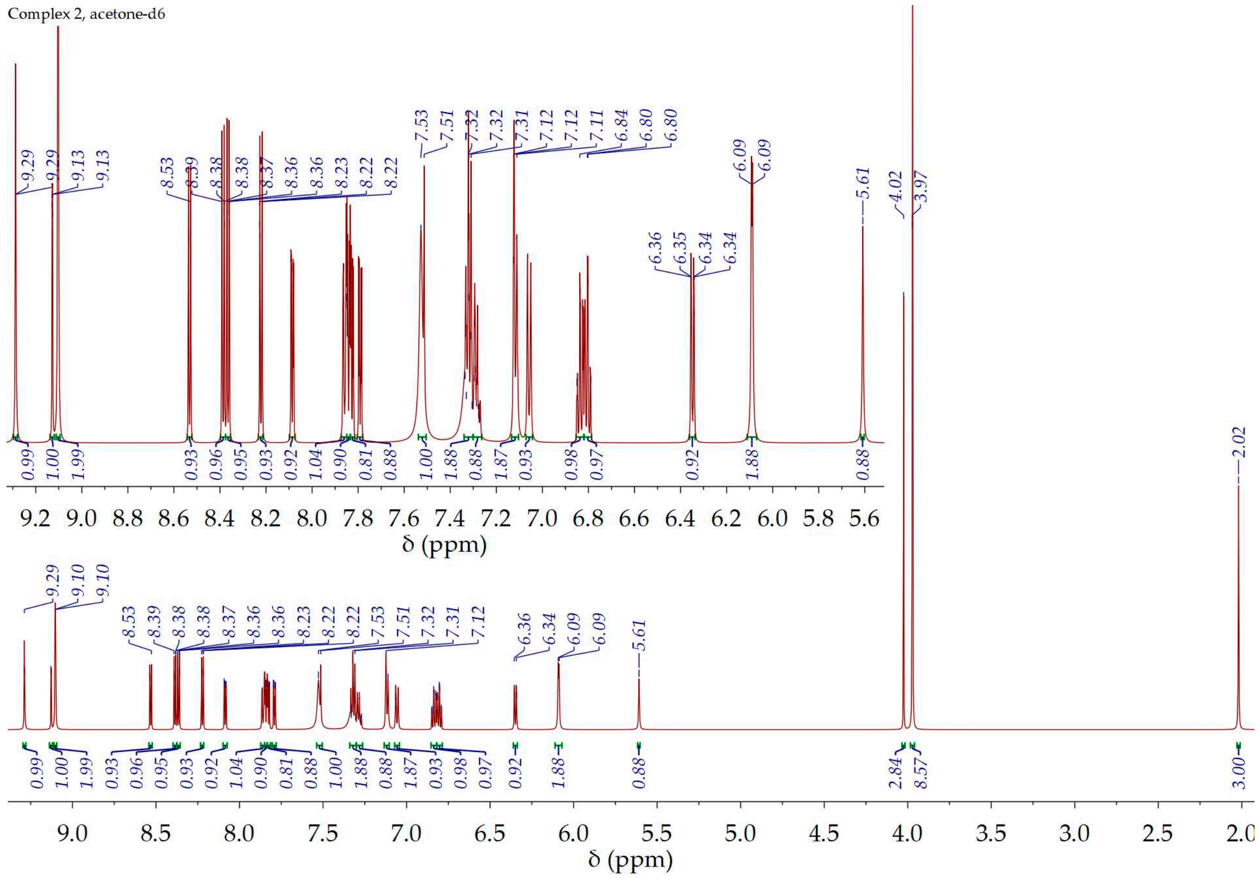

Figure S14. ^{1}H NMR spectrum of **2** (600 MHz, 298 K, acetone-d^6).

^{1}H NMR (600 MHz, Acetone-*d*$_6$) δ 9.29 (d, *J* = 0.9 Hz, 1H), 9.13 (d, *J* = 1.3 Hz, 1H), 9.10 (d, *J* = 1.7 Hz, 2H), 8.53 (dd, *J* = 5.7, 0.7 Hz, 1H), 8.39 (dd, *J* = 6.0, 0.6 Hz, 1H), 8.36 (dd, *J* = 5.9, 0.6 Hz, 1H), 8.22 (dd, *J* = 6.0, 0.6 Hz, 1H), 8.09 (dd, *J* = 5.6, 1.6 Hz, 1H), 7.86 (dd, *J* = 7.8, 1.1 Hz, 1H), 7.84 (dd, *J* = 6.0, 1.8 Hz, 1H), 7.83 (dd, *J* = 6.0, 1.8 Hz, 1H), 7.79 (dd, *J* = 6.0, 1.8 Hz, 1H), 7.54 – 7.50 (m, 1H), 7.34 – 7.30 (m, 2H), 7.30 – 7.26 (m, 1H), 7.13 – 7.10 (m, 2H), 7.06 (dd, *J* = 8.4, 1.0 Hz, 1H), 6.84 (td, *J* = 7.5, 1.5 Hz, 1H), 6.80 (td, *J* = 7.3, 1.4 Hz, 1H), 6.35 (dd, *J* = 7.3, 1.2 Hz, 1H), 6.09 (d, *J* = 3.2 Hz, 2H), 5.61 (s, 1H), 4.02 (s, 3H), 3.98 – 3.96 (m, 9H), 2.02 (s, 3H).

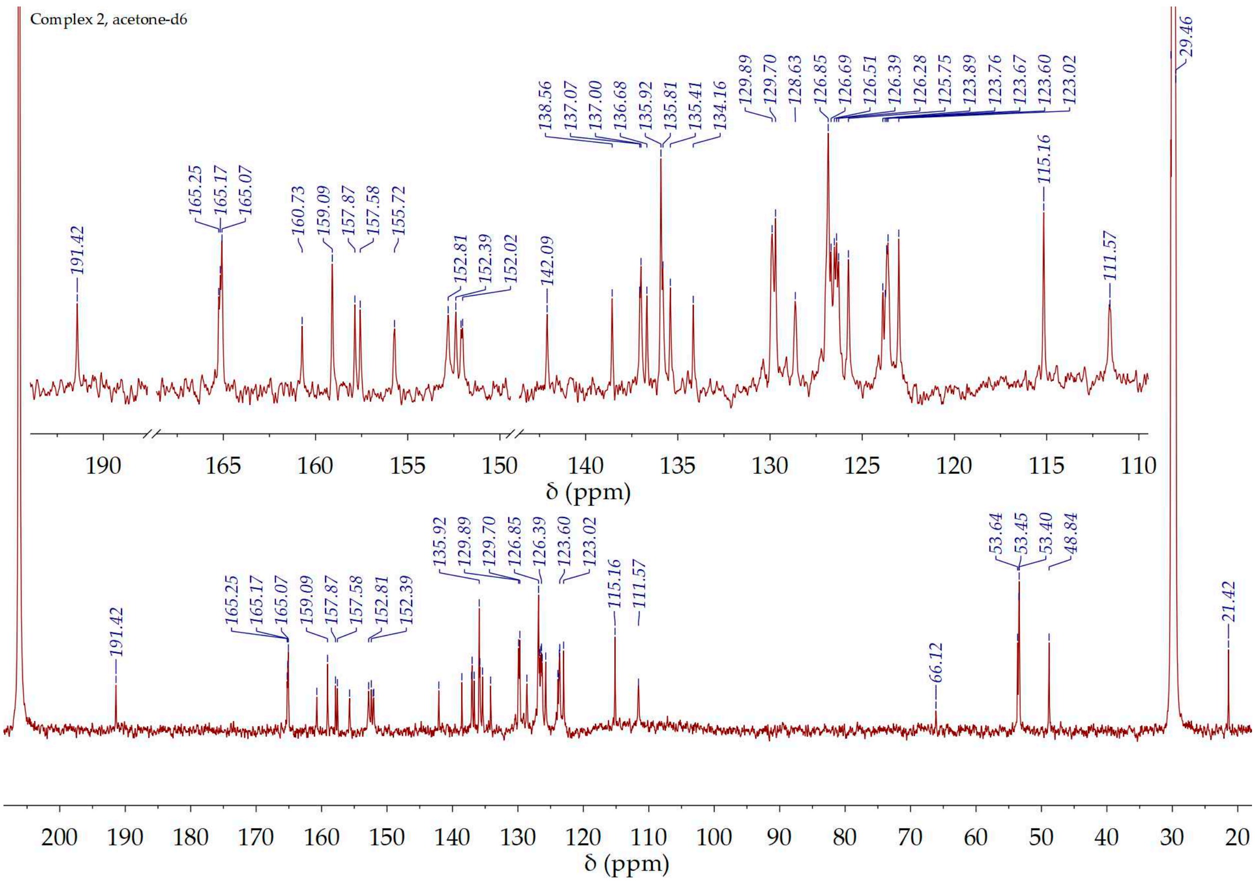

Figure S15. ^{13}C NMR spectrum of **2** (151 MHz, 298 K, acetone-d^6).

^{13}C NMR (151 MHz, acetone-d_6) δ 191.42, 165.25, 165.17, 165.07, 165.07, 160.73, 159.09, 159.09, 157.87, 157.58, 155.72, 152.81, 152.39, 152.11, 152.02, 142.09, 138.56, 137.07, 137.00, 136.68, 135.92, 135.92, 135.81, 135.41, 134.16, 129.89, 129.70, 128.63, 126.85, 126.85, 126.69, 126.51, 126.39, 126.28, 125.75, 123.89, 123.76, 123.67, 123.60, 123.02, 115.16, 111.57, 66.12, 53.64, 53.45, 53.40, 53.40, 48.84, 21.42.

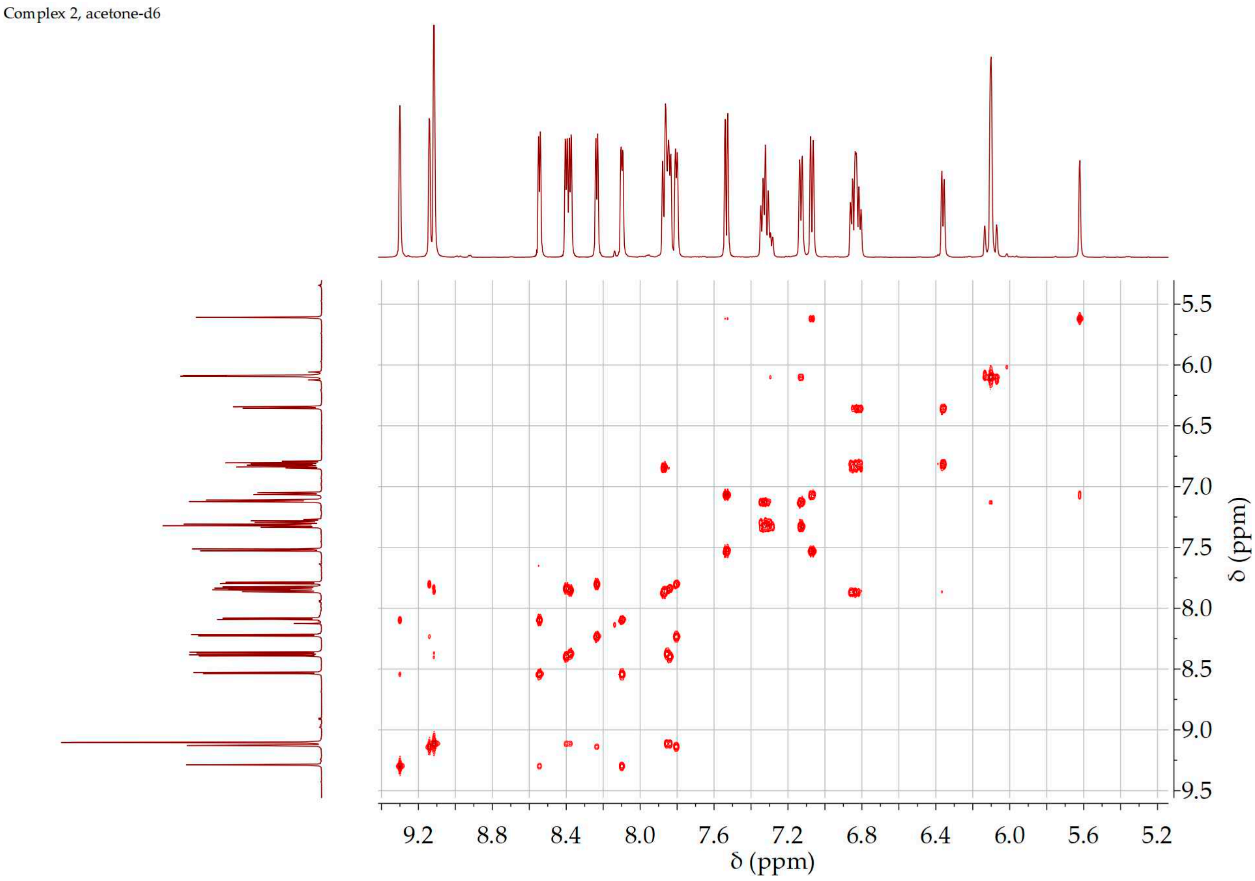

Figure S16. Aromatic region of COSY ^{1}H,^{1}H spectrum of **2**.

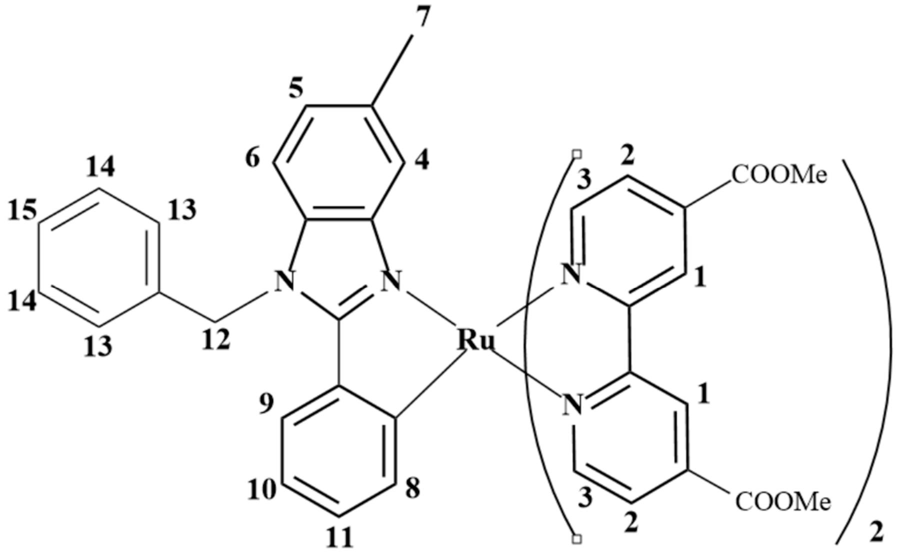

Table S5. Assignment of ^{1}H signals of complex **2**.

Hydrogen	^{1}H δ, ppm
**1**	9.29 (d, J = 0.9 Hz, 1H), 9.13 (d, J = 1.3 Hz, 1H), 9.10 (d, J = 1.7 Hz, 2H)
**2**	8.53 (dd, J = 5.7, 0.7 Hz, 1H), 8.39 (dd, J = 6.0, 0.6 Hz, 1H), 8.36 (dd, J = 5.9, 0.6 Hz, 1H), 8.22 (dd, J = 6.0, 0.6 Hz, 1H)
**3**	8.09 (dd, J = 5.6, 1.6 Hz, 1H), 7.84 (dd, J = 6.0, 1.8 Hz, 1H), 7.83 (dd, J = 6.0, 1.8 Hz, 1H), 7.79 (dd, J = 6.0, 1.8 Hz, 1H)

**4**	5.61 (s, 1H)
**5**	7.06 (dd, J = 8.4, 1.0 Hz, 1H)
**6**	7.54 – 7.50 (m, 1H)
**7**	2.02 (s, 3H)
**8**	6.35 (dd, J = 7.3, 1.2 Hz, 1H)
**9**	7.86 (dd, J = 7.8, 1.1 Hz, 1H)
**10**	6.84 (td, J = 7.5, 1.5 Hz, 1H)
**11**	6.80 (td, J = 7.3, 1.4 Hz, 1H)
**12**	6.09 (d, J = 3.2 Hz, 2H)
**13**	7.13 – 7.10 (m, 2H)
**14**	7.34 – 7.30 (m, 2H)
**15**	7.30 – 7.26 (m, 1H)
**COOMe**	4.02 (s, 3H), 3.98 – 3.96 (m, 9H)

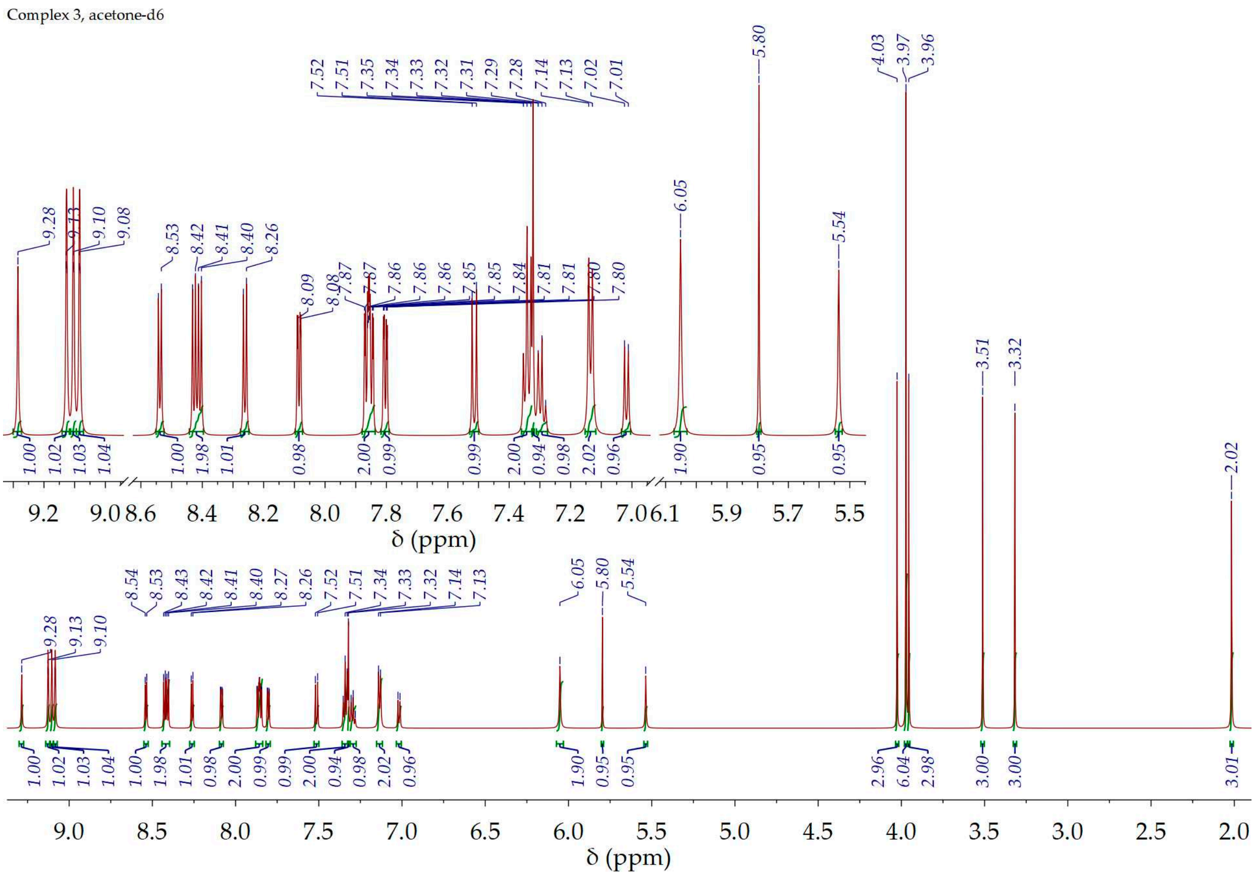

Figure S17. ^{1}H NMR spectrum of **3** (600 MHz, 298 K, acetone-d^{6}).

^{1}H NMR (600 MHz, Acetone-d_6) δ 9.28 (s, 1H), 9.13 (d, J = 1.3 Hz, 1H), 9.10 (d, J = 1.3 Hz, 1H), 9.08 (d, J = 1.3 Hz, 1H), 8.54 (d, J = 5.6 Hz, 1H), 8.43 (d, J = 5.9 Hz, 1H), 8.41 (d, J = 5.9 Hz, 1H), 8.26 (d, J = 6.0 Hz, 1H), 8.08 (dd, J = 5.6, 1.5 Hz, 1H), 7.88 – 7.84 (m, 2H), 7.80 (dd, J = 5.9, 1.7 Hz, 1H), 7.51 (d, J = 8.4 Hz, 1H), 7.34 (t, J = 7.3 Hz, 2H), 7.32 (s, 1H), 7.29 (t, J = 7.3 Hz, 1H) , 7.16 – 7.11 (m, 2H), 7.04 – 7.00 (m, 1H), 6.09 – 6.01 (m, 2H), 5.80 (s, 1H), 5.54 (s, 1H), 4.03 (s, 3H), 3.97 (s, 6H), 3.96 (s, 3H), 3.51 (s, 3H), 3.32 (s, 3H), 2.02 (s, 3H).

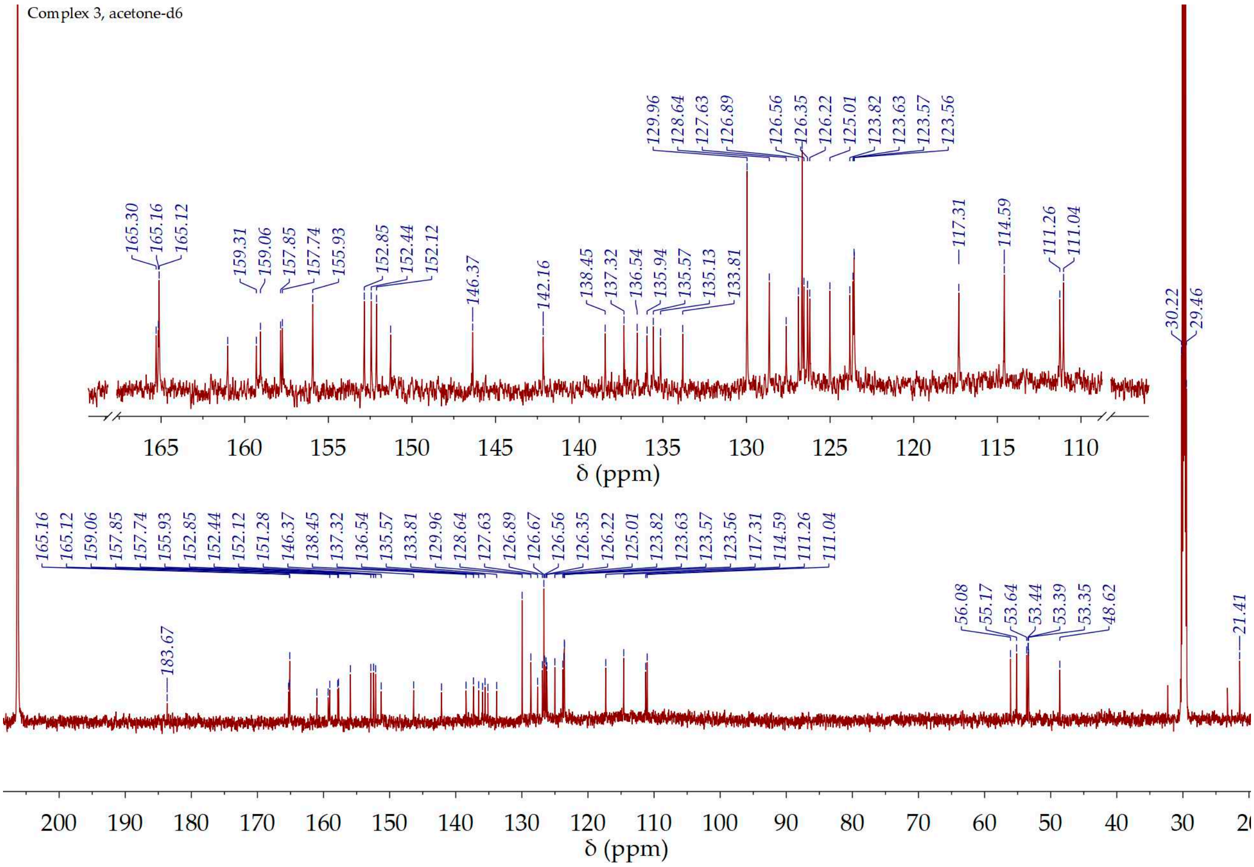

Figure S18. ^{13}C NMR spectrum of **3** (151 MHz, 298 K, acetone-d^6).

^{13}C NMR (151 MHz, Acetone) δ 183.67, 165.30, 165.16, 165.12, 165.12, 161.02, 159.31, 159.06, 157.85, 157.74, 155.93, 152.85, 152.44, 152.12, 151.28, 146.37, 142.16, 138.45, 137.32, 136.54, 135.94, 135.57, 135.13, 133.81, 129.96, 129.96, 128.64, 127.63, 126.89, 126.67, 126.67, 126.56, 126.35, 126.22, 125.01, 123.82, 123.63, 123.57, 123.56, 117.31, 114.59, 111.26, 111.04, 56.08, 55.17, 53.64, 53.44, 53.39, 53.35, 48.62, 21.41.

Complex 3, acetone-d6

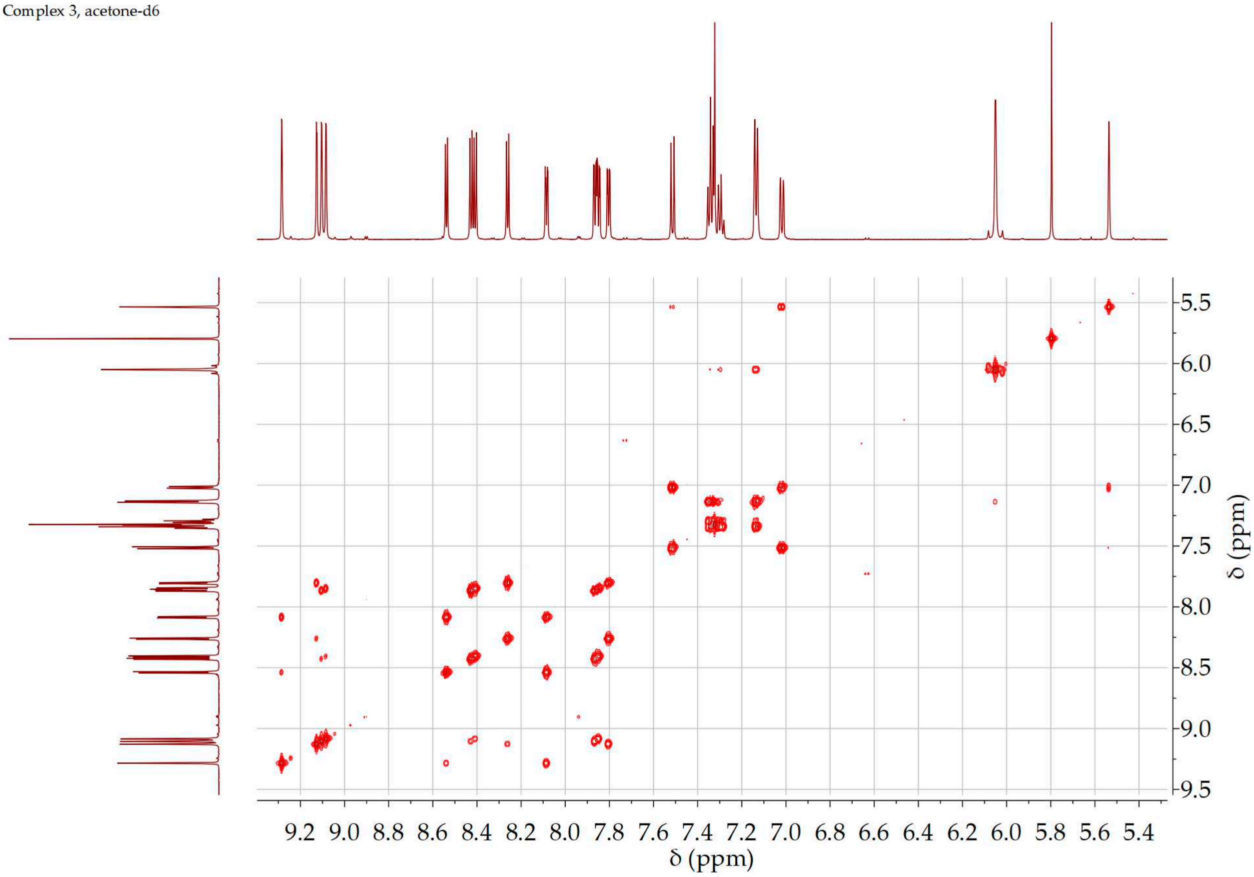

Figure S19. Aromatic region of COSY ^{1}H,^{1}H spectrum of **3**.

Complex 3, acetone-d6

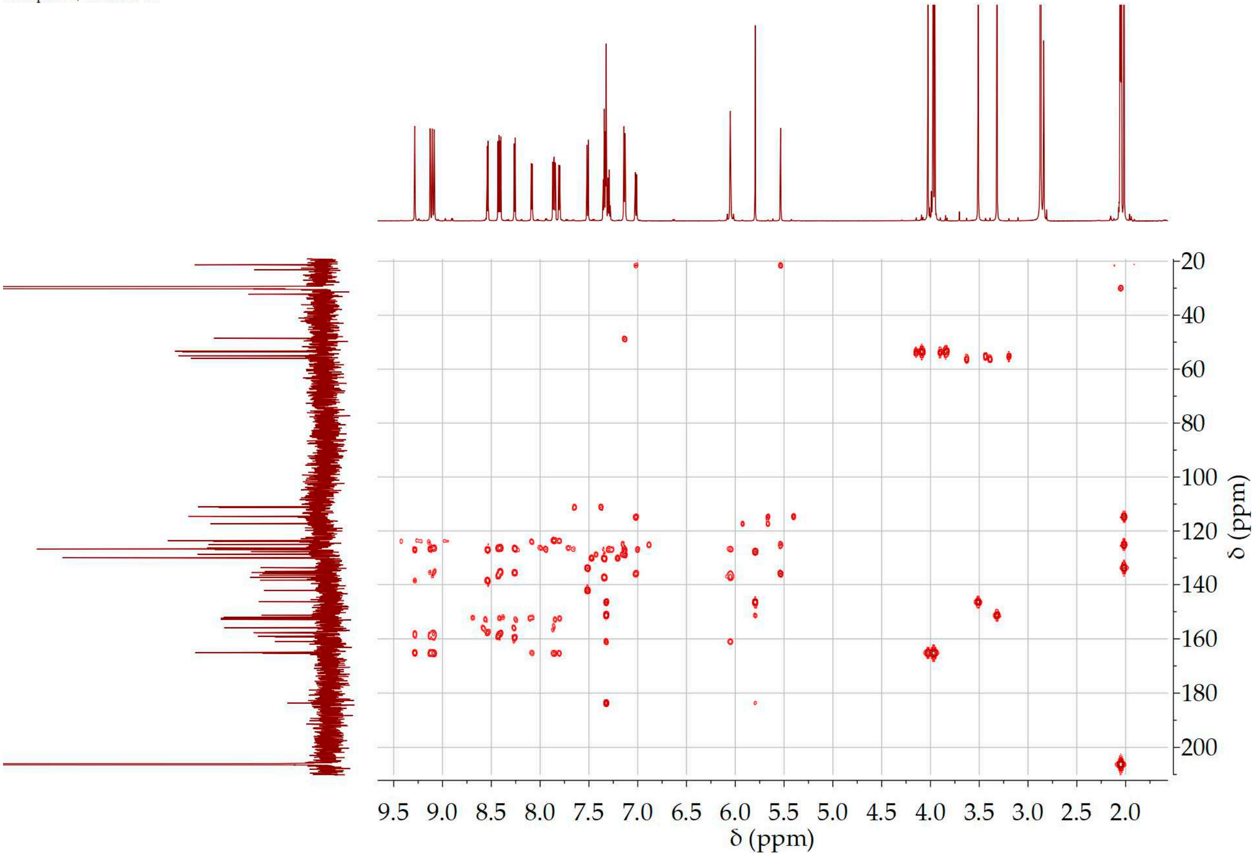

Figure S20. HMBC spectrum of **3**.

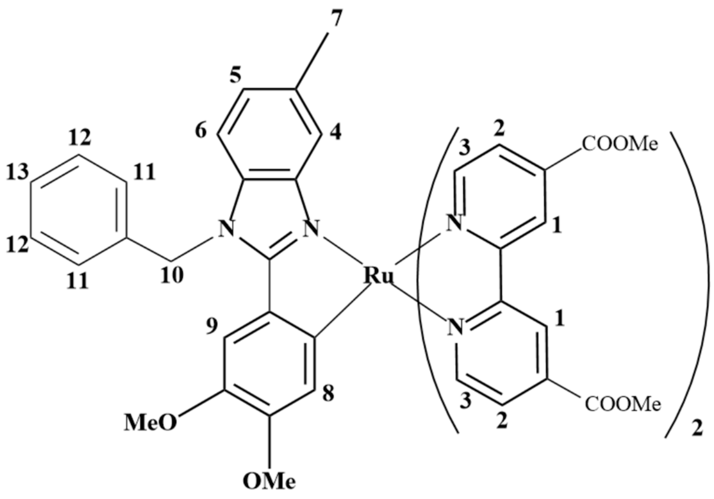

Table S6. Assignment of ^{1}H signals of complex **3**.

Hydrogen	^{1}H δ, ppm
**1**	δ 9.28 (s, 1H), 9.13 (d, *J* = 1.3 Hz, 1H), 9.10 (d, *J* = 1.3 Hz, 1H), 9.08 (d, *J* = 1.3 Hz, 1H)
**2**	8.54 (d, *J* = 5.6 Hz, 1H), 8.43 (d, *J* = 5.9 Hz, 1H), 8.41 (d, *J* = 5.9 Hz, 1H), 8.26 (d, *J* = 6.0 Hz, 1H)
**3**	8.08 (dd, *J* = 5.6, 1.5 Hz, 1H), 7.88 – 7.84 (m, 2H), 7.80 (dd, *J* = 5.9, 1.7 Hz, 1H)
**4**	5.54 (s, 1H)
**5**	7.04 – 7.00 (m, 1H)
**6**	7.51 (d, *J* = 8.4 Hz, 1H)
**7**	2.02 (s, 3H)
**8**	5.80 (s, 1H)
**9**	7.32 (s, 1H)
**10**	6.09 – 6.01 (m, 2H)
**11**	7.16 – 7.11 (m, 2H)
**12**	7.34 (t, *J* = 7.3 Hz, 2H)
**13**	7.29 (t, *J* = 7.3 Hz, 1H)
**OMe**	3.51 (s, 3H)
**OMe**	3.32 (s, 3H)
**COOMe**	4.03 (s, 3H), 3.97 (s, 6H), 3.96 (s, 3H)

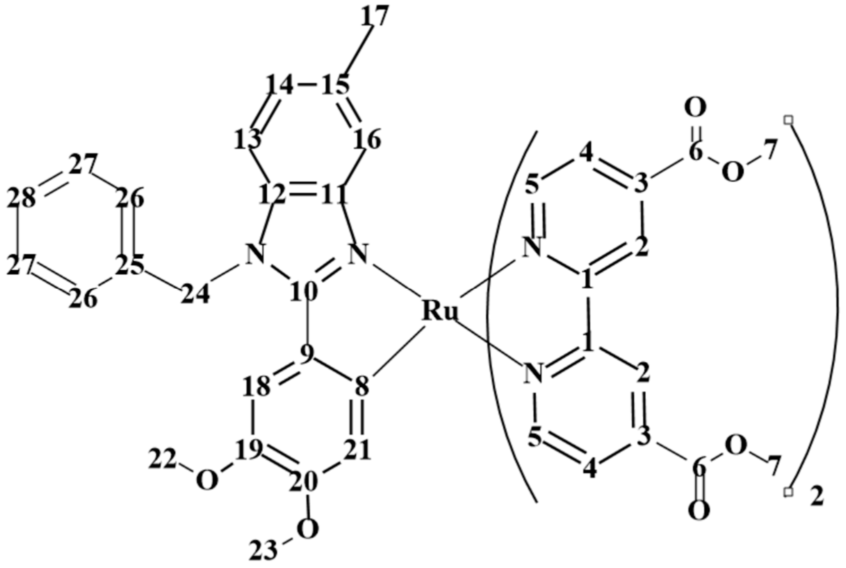

Table S7. Assignment of ^{13}C signals of complex **3**.

Carbon	^{13}C δ, ppm	^{1}H δ, ppm	Hydrogen
**1**	159.31, 159.06, 157.85, 157.74	-	**-**
**2**	123.82, 123.63, 123.57, 123.56	9.28, 9.13, 9.10, 9.08	**1**
**3**	138.45, 136.54, 135.57, 135.13	-	**-**
**4**	155.93, 152.85, 152.44, 152.12	8.54, 8.43, 8.41, 8.26	**2**
**5**	126.89, 126.56, 126.35, 126.22	8.08, 7.88 – 7.84, 7.80	**3**
**6**	165.30, 165.16, 165.12, 165.12	-	**-**
**7**	53.64, 53.44, 53.39, 53.35	4.03, 3.97, 3,96	**COOMe**
**8**	183.67	-	**-**
**9**	127.63	-	**-**
**10**	161.02	-	**-**
**11**	135.94	-	**-**
**12**	142.16	-	**-**
**13**	111.26	7.51	**6**
**14**	125.01	7.04 – 7.00	**5**
**15**	133.81	-	**-**
**16**	114.59	5.54	**4**
**17**	21.41.	2.02	**7**
**18**	111.04	7.32	**9**
**19**	146.37	-	**-**
**20**	151.28	-	**-**
**21**	117.31	5.80	**8**
**22**	56.08	3.51	**OMe**
**23**	55.17	3.32	**OMe**
**24**	48.62	6.09 – 6.01	**10**
**25**	137.32	-	**-**
**26**	126.67, 126.67	7.16 – 7.11	**11**
**27**	129.96, 129.96	7.34	**12**
**28**	128.64	7.29	**13**

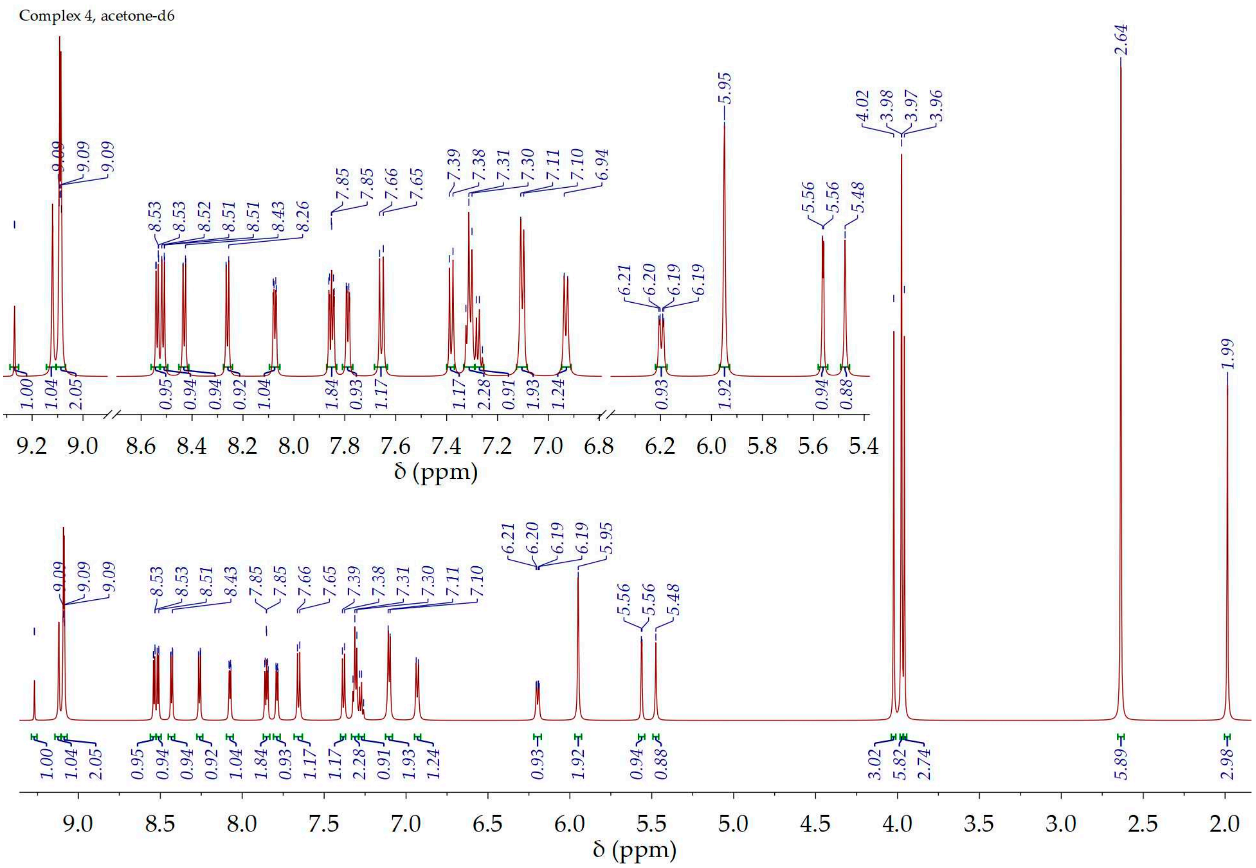

Figure S21. ^{1}H NMR spectrum of **4** (600 MHz, 298 K, acetone-d^{6}).

^{1}H NMR (600 MHz, Acetone-d_6) δ 9.27 (d, *J* = 1.2 Hz, 1H), 9.12 (d, 1H), 9.09 (d, 2H), 8.54 (dd, 1H), 8.51 (dd, 1H), 8.43 (dd, 1H), 8.26 (dd, 1H), 8.07 (dd, *J* = 5.7, 1.7 Hz, 1H), 7.87 – 7.83 (m, 2H), 7.79 (dd, *J* = 6.0, 1.8 Hz, 1H), 7.66 (d, *J* = 8.8 Hz, 1H), 7.38 (d, *J* = 8.3 Hz, 1H), 7.31 (t, *J* = 7.1 Hz, 2H), 7.27 (t, *J* = 7.3 Hz, 1H), 7.10 (d, *J* = 7.2 Hz, 2H), 6.93 (d, *J* = 8.0 Hz, 1H), 6.20 (dd, *J* = 8.9, 2.6 Hz, 1H), 5.95 (s, 2H), 5.56 (d, *J* = 2.6 Hz, 1H), 5.48 (s, 1H), 4.02 (s, 3H), 3.98 – 3.97 (m, 6H), 3.96 (s, 3H), 2.64 (s, 6H), 1.99 (s, 3H).

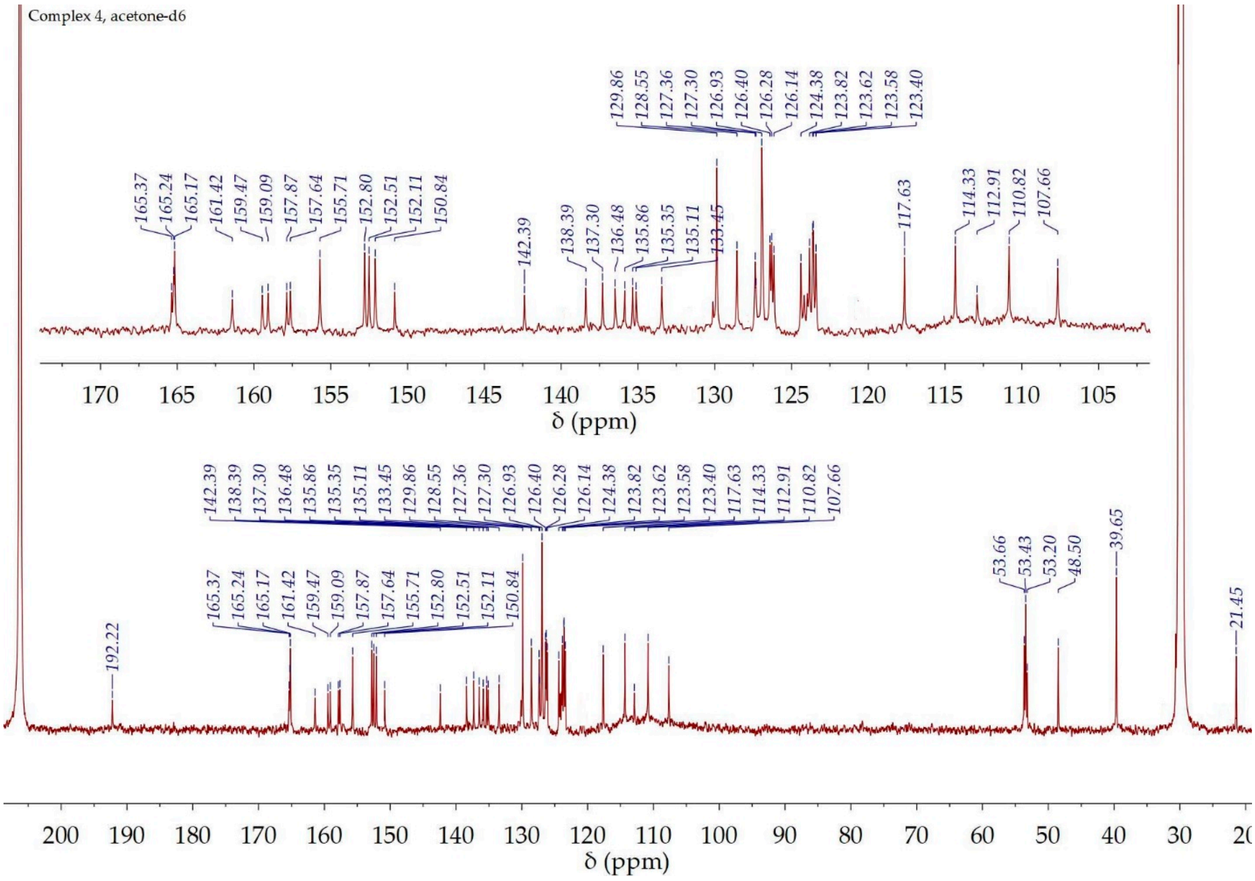

Figure S22. ^{13}C NMR spectrum of **4** (151 MHz, 298 K, acetone-d^6).

^{13}C NMR (151 MHz, Acetone) δ 192.22, 165.37, 165.24, 165.17, 165.17, 161.42, 159.47, 159.09, 157.87, 157.64, 155.71, 152.80, 152.51, 152.11, 150.84, 142.39, 138.39, 137.30, 136.48, 135.86, 135.35, 135.11, 133.45, 129.86, 129.86, 128.55, 127.36, 127.30, 126.93, 126.93, 126.40, 126.28, 126.14, 124.38, 123.82, 123.62, 123.58, 123.40, 117.63, 114.33, 112.91, 110.82, 107.66, 53.66, 53.43, 53.43, 53.20, 48.50, 39.65, 39.65, 21.45.

Complex 4, acetone-d6

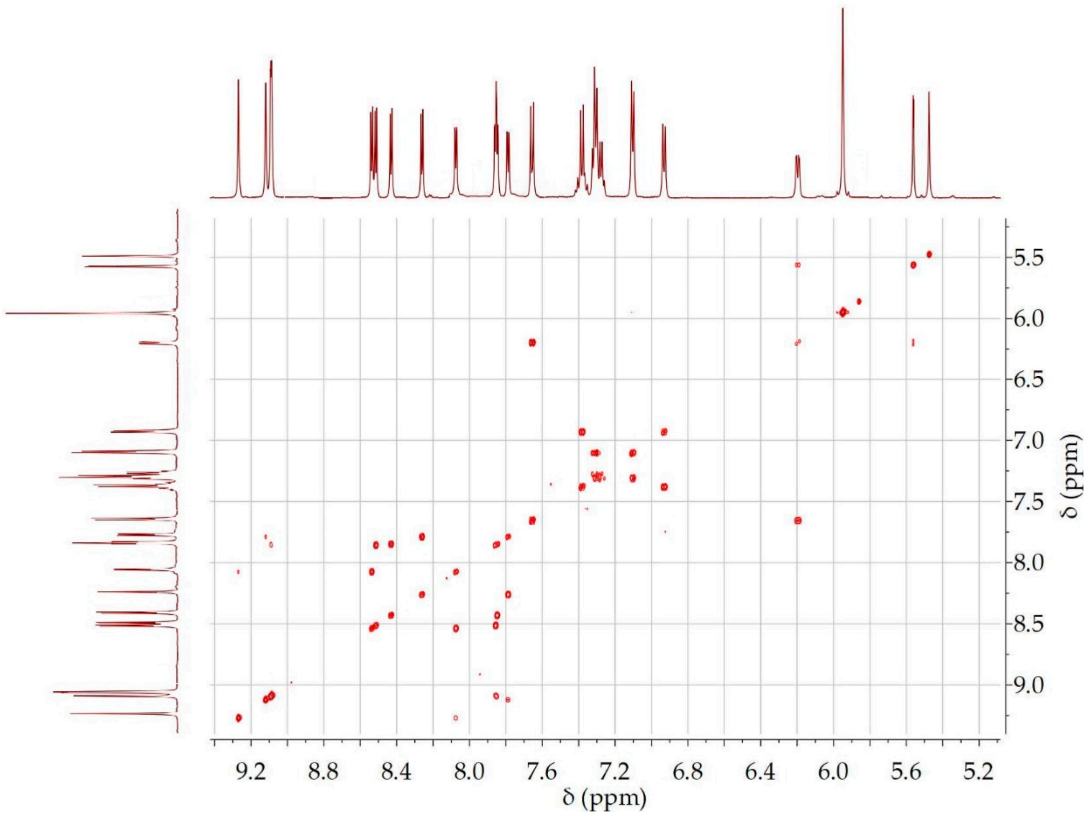

Figure S23. Aromatic region of COSY $^{1}H,^{1}H$ spectrum of **4**.

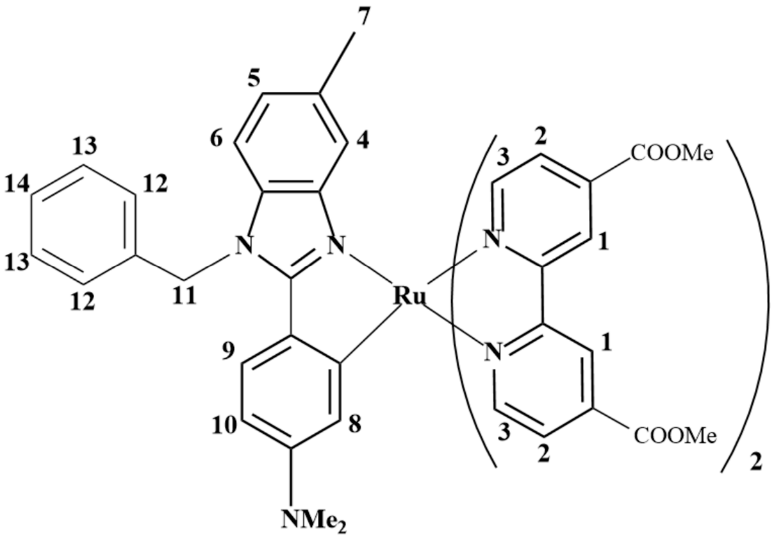

Table S8. Assignment of ^{1}H signals of complex **4**.

Hydrogen	^{1}H δ, ppm
**1**	9.27 (d, *J* = 1.2 Hz, 1H), 9.12 (d, 1H), 9.09 (d, 2H)
**2**	8.54 (dd, 1H), 8.51 (dd, 1H), 8.43 (dd, 1H), 8.26 (dd, 1H)
**3**	8.07 (dd, *J* = 5.7, 1.7 Hz, 1H), 7.87 – 7.83 (m, 2H), 7.79 (dd, *J* = 6.0, 1.8 Hz, 1H)
**4**	5.48 (s, 1H)
**5**	6.93 (d, *J* = 8.0 Hz, 1H)
**6**	7.38 (d, *J* = 8.3 Hz, 1H)

**7**	1.99 (s, 3H)
**8**	5.56 (d, *J* = 2.6 Hz, 1H)
**9**	7.66 (d, *J* = 8.8 Hz, 1H)
**10**	6.20 (dd, *J* = 8.9, 2.6 Hz, 1H)
**11**	5.95 (s, 2H)
**12**	7.10 (d, *J* = 7.2 Hz, 2H)
**13**	7.31 (t, *J* = 7.1 Hz, 2H)
**14**	7.27 (t, *J* = 7.3 Hz, 1H)
**NMe_2**	2.64 (s, 6H)
**COOMe**	4.02 (s, 3H), 3.98 – 3.97 (m, 6H), 3.96 (s, 3H)

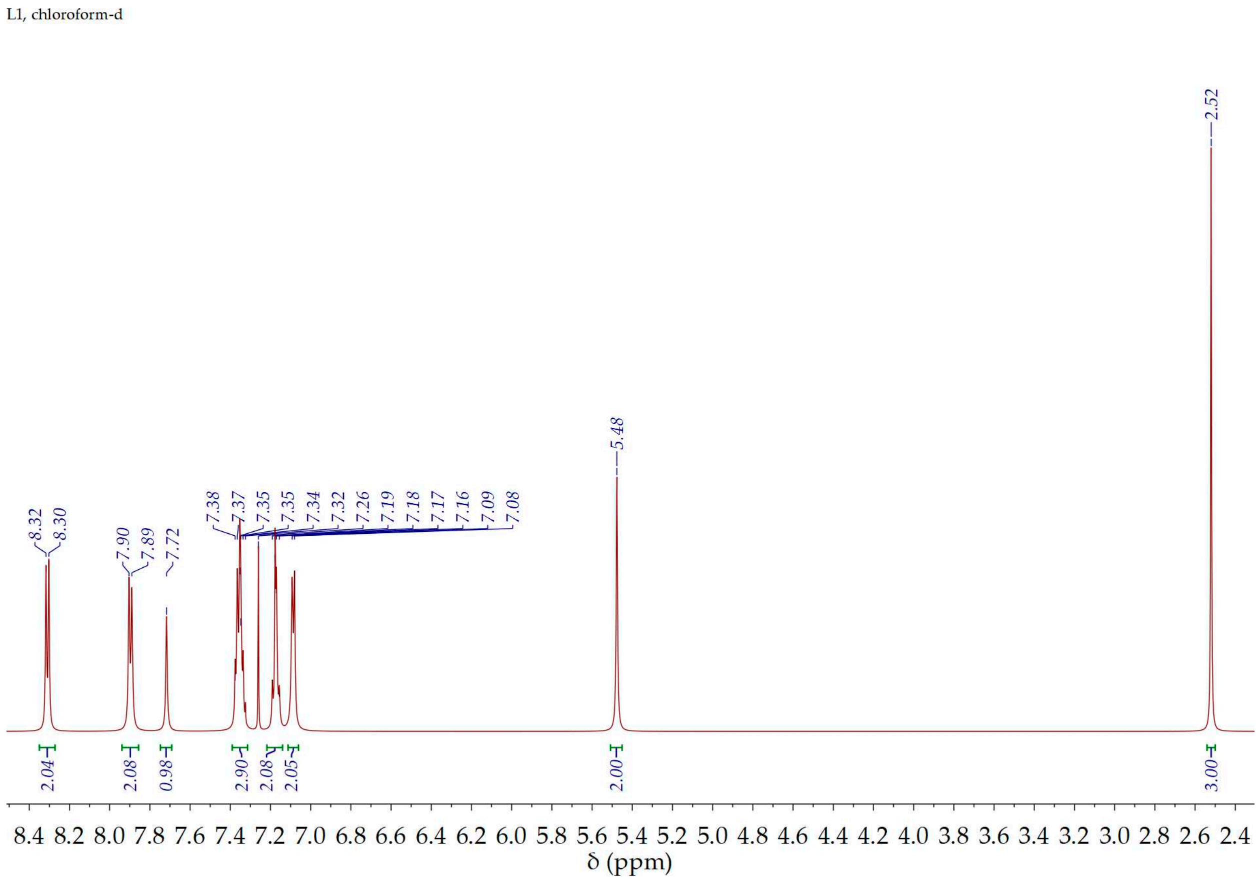

Figure S24. ^{1}H spectrum of **$L-NO_2$** (1-benzyl-2-(4-nitrophenyl)benzimidazole)

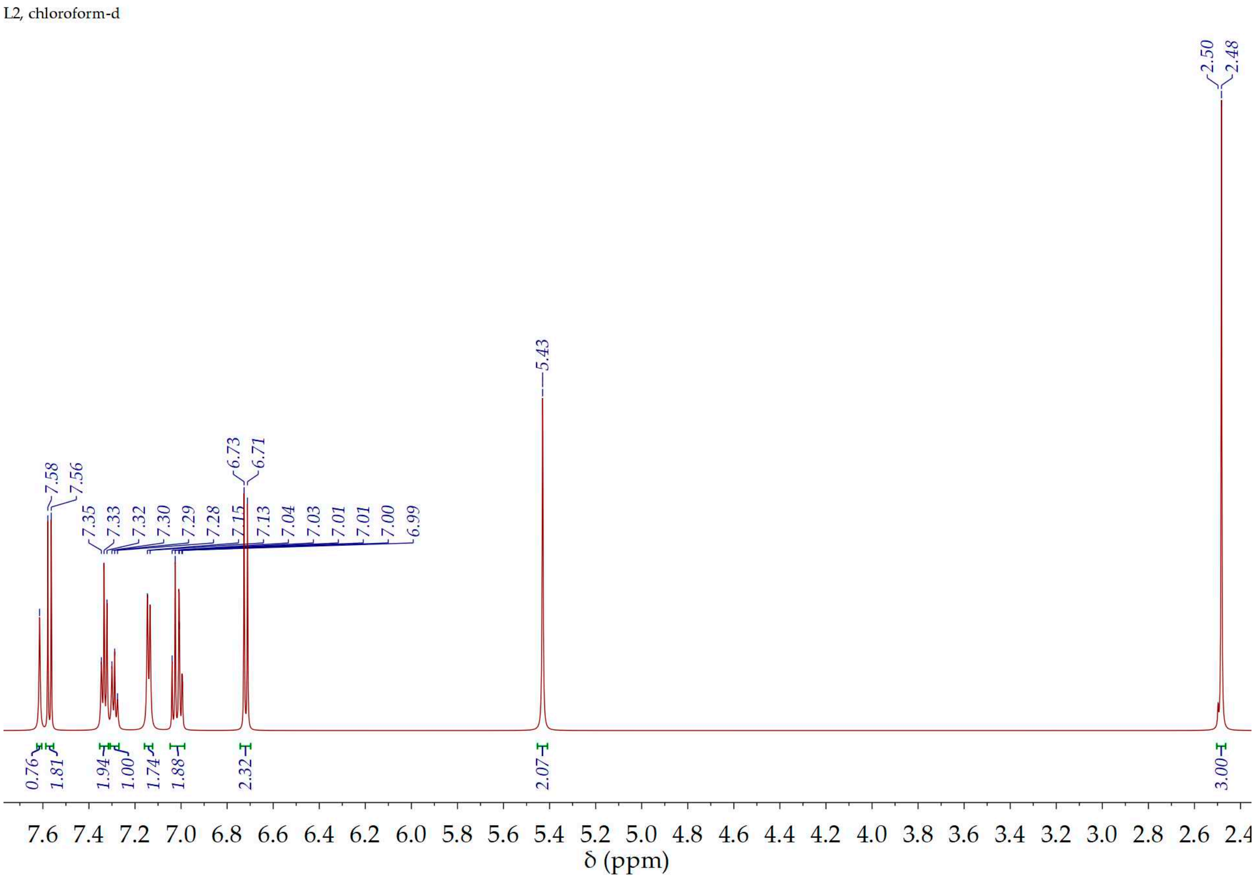

Figure S25. ^{1}H spectrum of **L-H** (1-benzyl-2-phenylbenzimidazole)

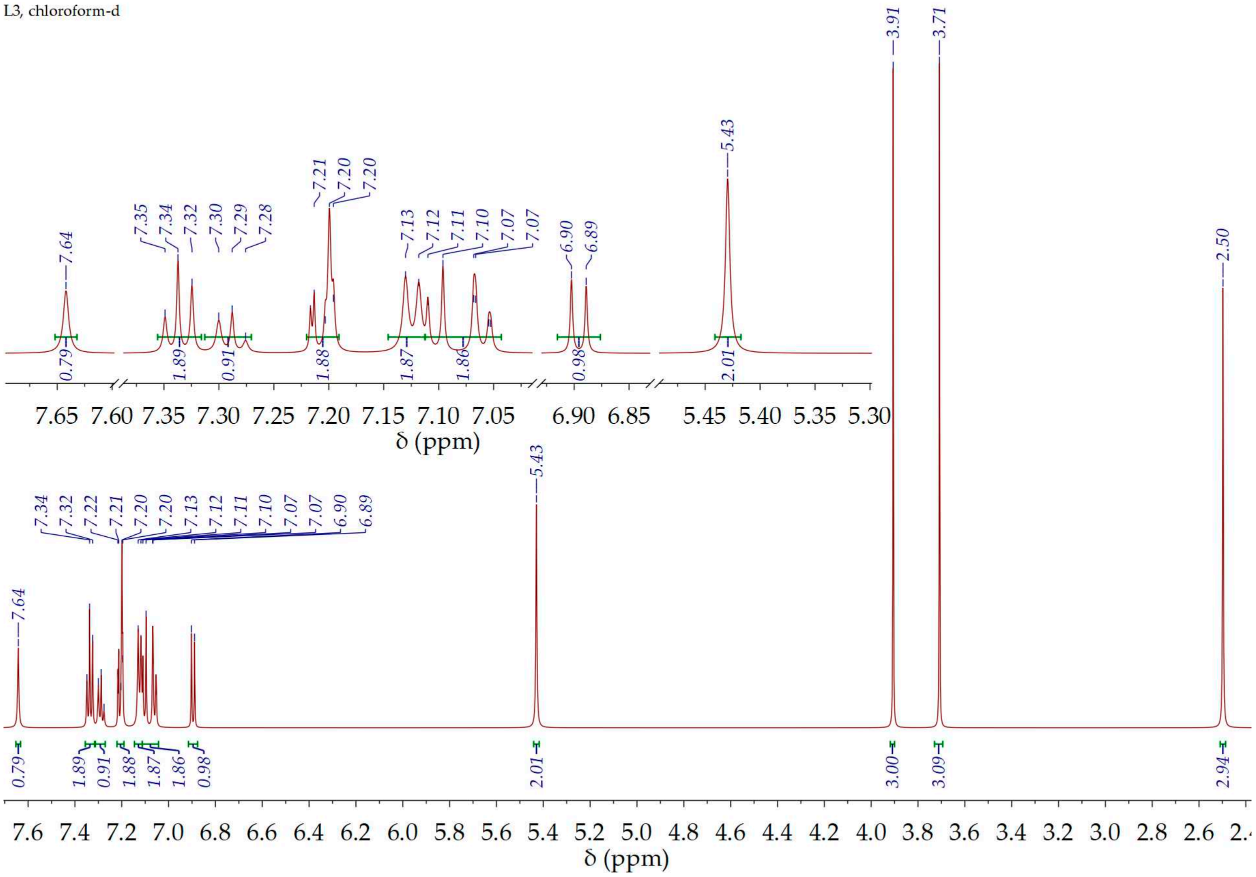

Figure S26. ^{1}H spectrum of **L-OMe$_2$** (1-benzyl-2-(3,4-dimethoxyphenyl)benzimidazole)

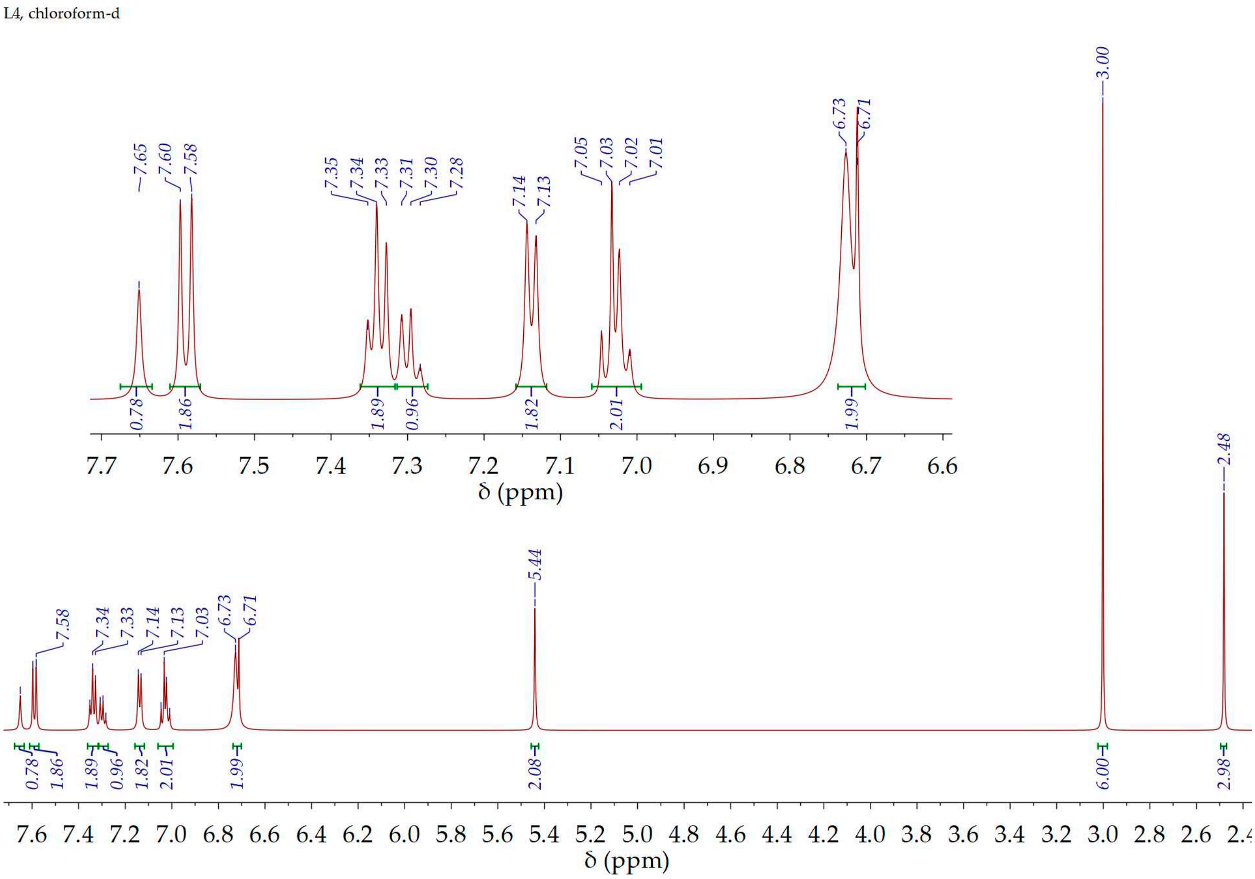

Figure S27. ^{1}H spectrum of **L-NMe₂** (1-benzyl-2-(4-dimethylaminophenyl)benzimidazole)
